# Nudix Hydrolase 13 Impairs the Initiation of Colorectal Cancer by Inhibiting PKM1 ADP‐Ribosylation

**DOI:** 10.1002/advs.202410058

**Published:** 2025-02-08

**Authors:** Jinlong Lin, Yixin Yin, Jinghua Cao, Bingxu Zou, Kai Han, Yufan Chen, Siyu Li, Cijun Huang, Jiewei Chen, Yongrui Lv, Shuidan Xu, Dan Xie, Fengwei Wang

**Affiliations:** ^1^ State Key Laboratory of Oncology in South China Guangdong Provincial Clinical Research Center for Cancer Sun Yat‐sen University Cancer Center Guangzhou 510060 P. R. China; ^2^ Department of Thoracic Surgery Sun Yat‐sen University Cancer Center Guangzhou 510060 P. R. China; ^3^ Department of Anesthesiology Sun Yat‐sen University Cancer Center Guangzhou 510060 P. R. China; ^4^ Department of Colorectal Surgery Sun Yat‐sen University Cancer Center Guangzhou 510060 P. R. China; ^5^ Department of Endoscopy Sun Yat‐sen University Cancer Center Guangzhou 510060 P. R. China; ^6^ Department of Pathology Sun Yat‐sen University Cancer Center Guangzhou 510060 P. R. China

**Keywords:** tumor initiation, CRC, ADP‐ribosylation, oxidative phosphorylation, PKM1, NUDT13

## Abstract

Metabolic dysregulation has been implicated as a key factor in colorectal cancer (CRC) initiation, however, the underlying driving forces and mechanisms remain poorly understood. Herein, transcriptome profiling of paired early‐stage CRCs and adenomas identifies Nudix hydrolase 13 (NUDT13) as a critical suppressor. Elevated NUDT13 expression impedes the proliferation of CRC cells under hypoxic conditions and markedly inhibits CRC initiation by upregulating PKM1. Mechanistically, NUDT13 directly binds and stabilizes PKM1 protein by reducing its poly ADP‐ribosylation (PARylation), which is catalyzed by PARP1 at E275/D281/E282/E285/D296, thereby inducing an oxidative phosphorylation (OXPHOS) phenotype in CRC cells. Moreover, spatiotemporal knockout of Nudt13 enhances intestinal tumorigenesis in mice, which can be significantly suppressed by PARP1 inhibitor Olaparib. Notably, residues E245/E248/E249 within the Nudix box motif of NUDT13 are essential for PKM1 PARylation, and a mimic peptide derived from this motif is sufficient to stabilize PKM1 protein and robustly inhibit CRC tumorigenesis. Collectively, this study reveals a previously unknown PARylation‐dependent mechanism that regulates PKM1 protein stability and switches the metabolic pathway of CRC cells, providing a promising target for CRC treatment.

## Graphical abstract

This study identified NUDT13 as a roadblock in the progression from colorectal adenoma to CRC, and revealed that NUDT13 stabilizes PKM1 protein by reducing the PARylation that is catalyzed by PARP1, thereby reprogramming the metabolic propensity of CRC cells. Moreover, a short peptide, mimicking the most conserved stretch of NUDT13, holds therapeutic effects in CRC.

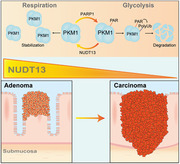

## 1 Introduction

Colorectal cancer (CRC) is a leading cause of cancer‐related mortality worldwide and is characterized by the sequential accumulation of mutations.^[^ ^1^ ^]^ Activating mutations in driver genes bestow adenomas with unlimited proliferative capacity, thereby instigating CRC.^[^ ^2^ ^]^ To meet the bioenergetic and biosynthetic needs for uncontrolled growth during CRC initiation, cancer cells undergo metabolic reprogramming, particularly under hypoxic and hypovascular conditions, to acquire a self‐sufficient phenotype, which includes enhanced glycolysis, nucleotide synthesis, and lipogenesis.^[^ ^1^, ^3^, ^4^, ^5^ ^]^ Among these, accelerated glycolysis represents the most fundamental feature of colorectal malignant transformation.^[^ ^6^, ^7^ ^]^ The high rate of glycolysis leads to the accumulation of glycolytic intermediates that are used as building blocks for macromolecular synthesis, thereby supporting tumor cell proliferation and reducing oxidative stress.^[^ ^4^ ^]^ Moreover, the shift from oxidative phosphorylation (OXPHOS) to glycolysis also intensifies the conversion of pyruvate to lactate, consequently acidifying the tumor microenvironment (TME) and enhancing the adaptability of tumor cells under stress conditions.^[^ ^8^ ^]^ However, the mechanisms bridging driver genes and metabolic dysregulation in CRC initiation remain unclear.

The nucleoside diphosphates linked to moiety‐X (Nudix) hydrolases are a ubiquitous class of enzymes across various species. These enzymes catalyze substrates dependent on the conserved Nudix box motif (Gx_5_Ex_5_[UA]xREx_2_EExGU), where U represents an aliphatic and hydrophobic residue.^[^ ^9^ ^]^ The founding member of the Nudix hydrolases family is MutT, a human NUDT1 homolog, and its first known function was to hydrolyze 8‐oxo‐dGTP to prevent its incorporation into nascent DNA.^[^ ^10^ ^]^ Because of this, the Nudix hydrolases family was initially defined as an important cellular security system, but have subsequently emerged as multifaceted enzymes with diverse substrates that include canonical and oxidized (d)NTPs, diadenosine polyphosphates, non‐nucleoside polyphosphates, and NAD‐RNAs.^[^ ^9^, ^11^ ^]^ Interestingly, dysregulated Nudix hydrolases have been detected in tumors under stress,^[^ ^12^, ^13^ ^]^ implying their potential importance for enabling tumor cells to adapt to adverse conditions. Therefore, elucidating the roles of Nudix hydrolases in cancer may hold compelling therapeutic prospects.

It is well‐established that 70–90% of CRCs arise from adenomas, yet only a small fraction progress to CRC.^[^ ^14^ ^]^ Identifying the metabolic regulators that drive the transition from adenomas to malignancies is crucial. In this study, we analyzed the transcriptome profiles of para‐tumors, adenomas, and early‐stage CRC tissues. Our results revealed that NUDT13, the most downregulated gene in early‐stage CRCs, plays a pivotal role in suppressing CRC initiation. NUDT13 stabilizes PKM1 protein, thereby redirecting metabolism from glycolysis toward OXPHOS. Notably, a small peptide derived from a conserved stretch of NUDT13 was sufficient to inhibit CRC initiation and growth, signifying NUDT13 as a promising target for therapeutic exploitation in CRC.

## 2 Results

### 2.1 NUDT13 Impedes the Tumorigenesis of CRC

**Figure 1:**
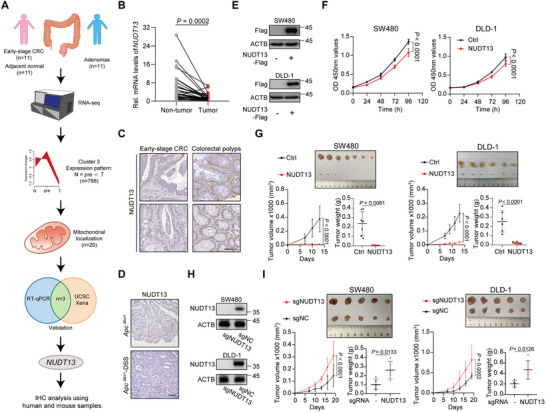
Downregulation of NUDT13 drives the initiation and progression of CRC. A) Identification of the potential key metabolic regulators driving the initiation of CRC. T: tumor. Pre: the precancerous lesions. N: normal. B) RT‐qPCR detection of NUDT13 mRNA expression in early‐stage CRC and paired normal tissues (*n* = 36), normalized to ACTB. C) IHC detection of NUDT13 in human early‐stage CRCs and precancerous polyps. Scale bars, 100 µm. D) IHC detection of NUDT13 in mouse CRCs (*Apc^Min/+^*‐DSS) and adenomas (*Apc^Min/+^*). Scale bars, 100 µm. E) Immunoblot analysis of NUDT13 protein levels after stable transfection of NUDT13 in SW480 and DLD‐1 cells. F) CCK8 assays measuring the proliferation rates of SW480 and DLD‐1 cells after stable transfection of NUDT13 or control vector. G) Subcutaneous tumors of nude mice with SW480 and DLD‐1 cells stably‐expressing control vector or NUDT13 (*n* = 6 or 7). Subcutaneous tumors were measured by volume and weight. H) Immunoblot analysis of NUDT13 protein levels after NUDT13 knockout in SW480 and DLD‐1 cells. I) Subcutaneous tumors of nude mice with NUDT13 knockout SW480 and DLD‐1 cells (*n* = 5). Subcutaneous tumors were measured by volume and weight. All results mentioned above were obtained from 3 or more independent experiments. Data are presented as mean ± SD; *P* values were calculated by Student's *t‐*test (B, G, and I) and two‐way ANOVA (F, G, and I).

To identify the potential key metabolic regulators driving the initiation of CRC, we analyzed the transcriptome profiles of colon adenomas from 11 individuals, stage I CRC tissues, and their corresponding para‐tumor tissues from an additional 11 patients (**Figure** **1**A). After clustering analysis, we classified the altered genes into 9 distinct expression patterns (Figure S1A, Supporting Information). Within these patterns, genes in clusters 1 and 5 showed consistent alterations that paralleled disease progression, suggesting their involvement in adenoma formation. In contrast, genes within clusters 2, 7, and 9 displayed a transition at the precancer stage, indicating their distinct roles during CRC tumorigenesis. Notably, clusters 3 and 8 exhibited a sharp change from precancer to cancer, implying that their dysregulation may drive the key step of malignant transformation. Here, we focused on cluster 3 and identified 20 genes with mitochondrial localization (Figure 1A; Table S1, Supporting Information). Among them, 3 genes (*NUDT13*, *PYROXD2*, and *YPEL3*) were validated through RT‐qPCR using a cohort comprising 36 paired early‐stage CRC samples and 14 paired adenomas, as well as through datasets from UCSC Xena (Figure 1B; Figure S2A–C, Supporting Information). NUDT13, a nucleoside diphosphate linked to moiety‐X (Nudix) hydrolase, emerged as the most significantly downregulated gene and therefore, was chosen for further study (Figure 1B). Further analysis using immunohistochemistry (IHC) confirmed the results of the screening (Figure 1C).

To investigate the involvement of NUDT13 in the transition from intestinal adenoma to carcinoma, we examined NUDT13 levels in the *Apc ^Min/+^*‐dextran sodium sulfate (DSS) mouse model, a system in which intestinal polyps progress to CRC upon DSS challenge. As shown in Figure 1D, NUDT13 was significantly downregulated in cancerous tissues compared to polyps, suggesting a conserved and important role of NUDT13 in CRC initiation. Further IHC analysis in a cohort of 174 clinical CRC cases revealed a negative correlation between NUDT13 levels and both T status and clinical stage (Table S2, Supporting Information). Multivariate analysis demonstrated that a low level of NUDT13 was an independent prognostic factor for poor survival in CRC patients (Table S3, Supporting Information). These results collectively support the role of NUDT13 in the initiation of CRC.

Next, we generated stable NUDT13‐overexpressing cells in SW480 and DLD‐1 (Figure 1E). In vitro functional assays revealed that NUDT13 overexpression resulted in a mild decrease in cell proliferation (Figure 1F; Figure S2D, Supporting Information). However, in subcutaneous xenograft models, NUDT13 overexpression sharply suppressed the tumor growth and tumorigenic potential of CRC cells (Figure 1G), and the knockout of NUDT13 accelerated xenograft tumor growth (Figure 1H,I). These results indicate that the in vivo microenvironment might contribute to the discrepancy in tumor progression.

### 2.2 NUDT13 Interacts with and Upregulates PKM1 Protein

**Figure 2:**
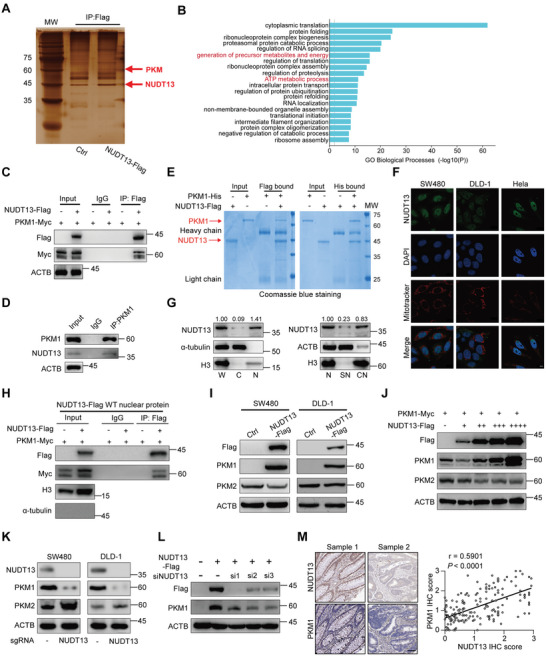
NUDT13 interacts with and upregulates PKM1. A) Detection of NUDT13‐binding proteins by silver stain following IP assays using protein extracts from the indicated DLD‐1 cells. B) GO analysis of potential NUDT13‐interacting proteins. (C and D) Co‐IP assays showed the interaction of NUDT13 with PKM1 in 293T C) and SW480 cells D). E) The direct interaction between NUDT13 and PKM1 was verified through in vitro reciprocal pull‐down assays using Coomassie blue staining. F) Representative immunofluorescence images showed the nuclear localization of NUDT13 in DLD‐1, SW480, and Hela cells. Scale bars, 10 µm. G) Left: subcellular protein fractionation indicated the nuclear localization of NUDT13 in SW480 cells. α‐tubulin and H3 were used as the controls for the cytoplasmic and nuclear proteins, respectively. Right: further protein fractionation using nuclear extracts from SW480 cells. β‐actin and H3 were used as controls for the soluble nuclear and chromatin‐bound proteins, respectively. W: whole cell lysates. C: cytoplasmic contents. N: nuclear proteins. SN: soluble nuclear extracts. CN: chromatin‐bound nuclear proteins. H) Co‐IP of exogenous NUDT13‐Flag and PKM1‐Myc in nuclear extracts from 293T cells. Histone H3 was used as the nuclear control. I) Immunoblot analysis of PKM1 and PKM2 levels after overexpression of NUDT13 in SW480 cells (left) and DLD‐1 cells (right). J) Immunoblot analysis of PKM1 levels after co‐transfection of 239T cells with PKM1‐Myc and indicated doses of NUDT13‐Flag plasmids. K) Immunoblot analysis of PKM1 and PKM2 levels after knockout of NUDT13 in SW480 cells (left) and DLD‐1 cells (right). L) Immunoblot analysis of PKM1 levels after transfection of NUDT13‐overexpressing SW480 cells with siRNA specific for NUDT13. M) Left: representative IHC images of NUDT13 and PKM1 staining in serial CRC sections. Scale bars, 100 µm. Right: Positive correlation between NUDT13 and PKM1 protein levels in 174 CRC samples. All results mentioned above were obtained from 3 independent experiments. Data are presented as mean ± SD; *P* value was calculated by Pearson correlation analysis (M).

To elucidate the underlying mechanism of NUDT13 in CRC initiation, we performed mass spectrum (MS) analysis following NUDT13 immunoprecipitation (IP) to identify its potential partners (**Figure** **2**A). Interestingly, Gene Ontology (GO) analysis of candidates showed that the generation of precursor metabolites and energy, and the ATP metabolic process were among the most enriched pathways (Figure 2B). Several glycolytic enzymes such as ENO1, LDHA, and PGK1, were identified in both pathways (Figure S3A,B, Supporting Information). Based on its dramatic role in glucose metabolism and tumorigenesis, we selected Pyruvate kinase M (PKM), a rate‐limiting enzyme in glycolysis, for further investigation. Reciprocal Co‐IP and pull‐down assays substantiated the direct interaction between NUDT13 and PKM1, but not PKM2 (Figure 2C–E; Figure S3C, Supporting Information).

Despite the initial prediction of mitochondrial localization, our data revealed that NUDT13 is located primarily in the nucleus (Figure 1C,D; Figure S3D, Supporting Information), a finding further supported by the presence of a nuclear localization signal (NLS) sequence (Figure S3E,F, Supporting Information). Consistently, both immunofluorescence detection and subcellular protein fractionation supported that human NUDT13 was exclusively located in the nucleus, and more exactly, mainly in the chromatin‐bound part (Figure 2F,G). Moreover, our co‐IP experiments using proteins extracted from the nucleus further confirmed the interaction between NUDT13 and PKM1 (Figure 2H).

Meanwhile, we found that the protein levels of PKM1, but not PKM2, were significantly enhanced in NUDT13‐stably transfected cells (Figure 2I), and that NUDT13 exhibited a dose‐dependent upregulation of PKM1 (Figure 2J). Conversely, the knockout or knockdown of NUDT13 decreased the PKM1 levels (Figure 2K; Figure S3G, Supporting Information). Furthermore, the upregulation of PKM1 induced by NUDT13 was reversed upon NUDT13 knockdown (Figure 2L; Figure S3H, Supporting Information). Interestingly, we observed that NUDT13 isoform 3, which lacks the 126 N‐terminal amino acids (AA), could bind to PKM1 in the nucleus and upregulate its protein levels (Figure S3I–K, Supporting Information). Next, we assessed the correlation between NUDT13 and PKM1 in CRC tissues. As expected, the levels of PKM1 were lower in CRC samples compared to their para‐tumor counterparts and exhibited a positive correlation with that of NUDT13 (Figure 2M). Additionally, low levels of PKM1 were positively correlated with aggressive clinicopathological characteristics (Table S4, Supporting Information). These results solidify the status of PKM1 as a downstream target of NUDT13.

### 2.3 NUDT13 Reprograms Glycometabolism of CRC Cells via PKM1

**Figure 3:**
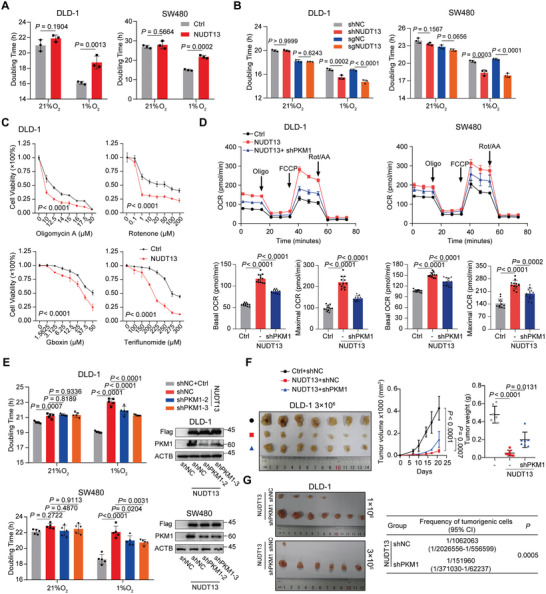
NUDT13 renders tumor cells addicted to oxygen for survival via PKM1. A) The doubling time of NUDT13‐overexpressing DLD‐1 and SW480 cells under normoxic (21%O_2_) or hypoxic (1%O_2_) conditions, as measured by CCK8. B) The doubling time of NUDT13 knockout or knockdown DLD‐1 and SW480 cells under normoxic (21%O_2_) or hypoxic (1%O_2_) conditions, as measured by CCK8. C) The cell viability of NUDT13‐overexpressing DLD‐1 cells treated with indicated concentrations of Oligomycin A, Rotenone, Gboxin, and Teriflunomide, as measured by CCK8. D) Top: the oxygen consumption rate (OCR) of SW480 cells and DLD‐1 cells transfected with the indicated vectors in response to oligomycin, FCCP, and rotenone/antimycin A. Bottom: bar graphs depicting the basal OCR (left) and the maximal OCR (right) of SW480 cells and DLD‐1 cells. E) Left: The doubling time of NUDT13‐overexpressing and PKM1 knockdown SW480 and DLD‐1 cells under normoxic or hypoxic conditions, as measured by CCK8. Right: immunoblot analysis of PKM1 levels to test the silencing effect of PKM1 shRNA in SW480 and DLD‐1 cells. F) Subcutaneous tumors of nude mice with DLD‐1 cells stably‐expressing the indicated vectors (*n* = 7). Subcutaneous tumors were measured by volume (left) and weight (right). G) Left: NUDT13‐overexpressing DLD‐1 cells transfected with or without shPKM1 were diluted and subcutaneously implanted into nude mice (*n* = 7). Right: frequency of tumorigenic cells. The tumorigenic cell frequency was estimated using an online tool at http://bioinf.wehi.edu.au/software/elda. All results mentioned above were obtained from 3 or more independent experiments. Data are presented as mean ± SD; *P* values were calculated by Student's *t‐*test (A, B, D, E, and F), two‐way ANOVA (C and F), and chi‐square test (G).

Studies have demonstrated that PKM1 promotes the conversion of pyruvate to acetyl‐CoA,^[^ ^15^, ^16^, ^17^, ^18^ ^]^ and that cells predominantly expressing PKM1 prefer OXPHOS for survival.^[^ ^15^, ^19^ ^]^ Based on this, we hypothesized that NUDT13 might exert its tumor‐suppressive effects by directing glycometabolism toward OXPHOS in CRC cells. Thus, we examined the proliferation rates of CRC cells under normoxic (21% O_2_) or hypoxic (1% O_2_) conditions. NUDT13 overexpression resulted in an extended doubling time under hypoxic but not normoxic conditions (**Figure** **3**A), whereas NUDT13 deficiency had the opposite effect (Figure 3B). Additionally, we also assessed whether other stresses commonly encountered in the TME, such as serum or glucose starvation, acidification, and oxidative stress,^[^ ^20^ ^]^ affect the phenotype of NUDT13 in CRC cells (Figure S4A,B, Supporting Information). Although 1% FBS slightly retarded the proliferation of NUDT13‐overexpressing cells, it failed to hinder their proliferation significantly compared to normal conditions (Figure S4C, Supporting Information). These results suggest that cells with elevated NUDT13 levels rely more on oxygen for survival and might be more sensitive to OXPHOS inhibitors. Therefore, we treated cells with electron transport chain (ETC) inhibitors, including oligomycin, rotenone, gboxin, and teriflunomide.^[^ ^21^, ^22^ ^]^ As shown in Figures 3C and S5A,B (Supporting Information), NUDT13‐overexpressing cells exhibited lower IC50 values for all of the ETC inhibitors, while NUDT13 depletion desensitized cells to these drugs (Figure S5C–E, Supporting Information). Moreover, Seahorse analysis provided further evidence that NUDT13 enhanced the oxygen consumption rate (OCR) while simultaneously inhibiting the extracellular acidification rate (ECAR) in CRC cells, indicating the glycometabolic reprogramming effect of NUDT13 (Figure 3D; Figure S5F–H, Supporting Information).

To explore whether PKM1 mediates the OXPHOS phenotype induced by NUDT13, we knocked down PKM1 in NUDT13‐stably transfected SW480 and DLD‐1 cells (Figure 3E, right). Rescue experiments showed that silencing PKM1 partially reversed the prolonged doubling time induced by NUDT13 under hypoxia (Figure 3E, left), while PKM1 overexpression in control cells displayed the opposite effect (Figure S6A–E, Supporting Information). Furthermore, PKM1 depletion enhanced the resistance of CRC cells to ETC inhibitors (Figure S6F–H, Supporting Information), whereas increased PKM1 sensitized cells to the ETC inhibitors (Figure S6I,J, Supporting Information). Consistently, both DLD‐1 and SW480 cells exhibited a preference for glucose oxidation upon PKM1 overexpression (Figure S6K,L, Supporting Information). In NUDT13‐overexpressing cells, the loss of PKM1 impeded NUDT13's ability to switch glycometabolism from aerobic glycolysis to OXPHOS (Figure 3D; Figure S5F, Supporting Information). Moreover, subcutaneous tumors in nude mice revealed that PKM1 knockdown restored the tumor growth and tumorigenic potential that had been suppressed by NUDT13 (Figure 3F,G).

We also noticed a slight reduction in the PKM2 protein levels upon exogenous expression of NUDT13 (Figure 2I). As PKM2 has been shown to strongly promote tumor progression in various cancers,^[^ ^23^, ^24^, ^25^ ^]^ we sought to investigate whether the downregulation of PKM2 contributes to the phenotype of NUDT13 and observed that NUDT13 had no significant effect on the expression of key downstream targets of PKM2 (Figure S6M, Supporting Information). These results provide further evidence that the NUDT13‐driven antitumor activity is primarily mediated through upregulation of PKM1, rather than downregulation of PKM2.

### 2.4 The NH Domain of NUDT13 is Essential for Binding to PKM1

**Figure 4:**
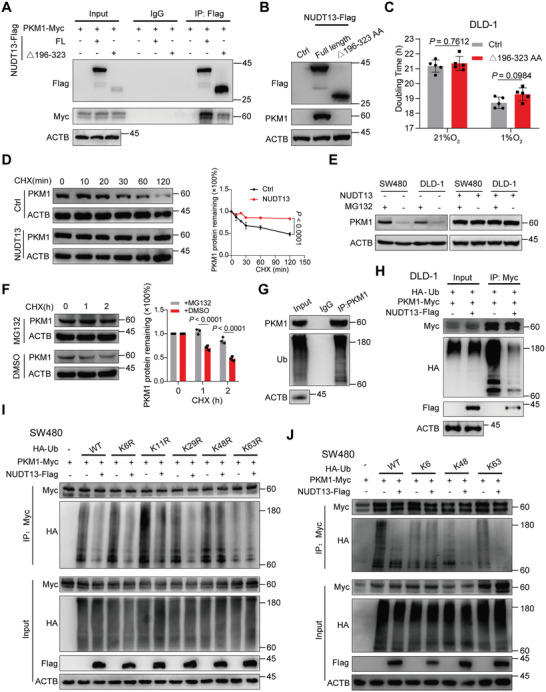
NUDT13 prevents the degradation of PKM1 by reducing its K48‐ and K63‐linked ubiquitin chains. A) Co‐IP assays were conducted in 293T cells co‐transfected with PKM1‐Myc and NUDT13‐Flag WT or mutant plasmids. B) Immunoblot analysis of the effect of a NUDT13 truncated mutant on PKM1 protein levels in DLD‐1 cells. C) The doubling time of DLD‐1 cells transfected with a NUDT13 truncated mutant or control vector under normoxic or hypoxic conditions, as measured by CCK8. D) Control and NUDT13‐overexpressing SW480 cells were exposed to 50µg mL^−1^ cycloheximide (CHX) for the indicated time. Quantification of PKM1 levels by densitometry. E) Immunoblot analysis of PKM1 levels after treatment with MG132 (10 µm, 4h) in NUDT13‐overexpressing or control SW480 and DLD‐1 cells. F) SW480 cells were co‐treated with CHX (50µg mL^−1^) and MG132 (10 µm). Quantification of PKM1 by densitometry. G) Immunoblot analysis of endogenous PKM1 ubiquitination levels in DLD‐1 cells. H) Immunoblot analysis of PKM1 ubiquitination levels after upregulation of NUDT13 in DLD1 cells transfected with PKM1‐Myc and HA‐Ub. I,J) Immunoblot analysis of PKM1 ubiquitin linkage types that were affected by NUDT13 in SW480 cells transfected with PKM1‐Myc, NUDT13‐Flag, and HA‐Ub WT or the ubiquitin mutants (K6R, K11R, K29R, K48R, K63R, K6, K48 or K63). All results mentioned above were obtained from 3 or more independent experiments. Data are presented as mean ± SD; *P* values were calculated by Student's *t‐*test (C) and two‐way ANOVA (D,F).

Next, we explored how NUDT13 interacts with PKM1. Given that NUDT13 isoform 3 was able to bind to PKM1, we constructed truncated NUDT13 mutants starting with residue 127 in order to investigate which domain interacts with PKM1 (Figure S7A, Supporting Information). Co‐IP experiments revealed that the Nudix hydrolase (NH) domain of NUDT13 was primarily responsible for its interplay with the 241–408 AA region of PKM1 (Figure S7B–E, Supporting Information). Furthermore, the removal of the NH domain of NUDT13 abolished its interaction with PKM1 (**Figure** **4**A), and exogenous expression of NUDT13 lacking the NH domain failed to increase PKM1 protein levels (Figure 4B; Figure S7F, Supporting Information). Additionally, anaerobic culture revealed that deletion of the NH domain of NUDT13 yielded outcomes similar to those observed in the control group (Figure 4C; Figure S7G, Supporting Information). These data collectively confirm that the NH domain of NUDT13 is required for the physical interaction with PKM1 and its biological function.

### 2.5 NUDT13 Stabilizes PKM1 by Reducing its K48‐ and K63‐Linked Ubiquitin Chains

Next, we investigated the mechanism by which NUDT13 elevates PKM1 protein levels. We found that NUDT13 had little effect on *PKM1* mRNA levels (Figure S8A,B, Supporting Information), and therefore hypothesized that NUDT13 might regulate the stability of PKM1 protein. As expected, NUDT13 robustly prolonged the half‐life of PKM1 following cycloheximide (CHX) treatment in CRC cells (Figure 4D). Moreover, MG132 treatment increased PKM1 protein levels and significantly extended the half‐life of PKM1 (Figure 4E,F). However, in the presence of NUDT13, MG132 failed to yield a similar effect (Figure 4E, right), suggesting that NUDT13 inhibits the proteasomal degradation of PKM1. Furthermore, we observed that endogenous and exogenous PKM1 could be ubiquitinated in vivo (Figure 4G; Figure S8C, Supporting Information), and NUDT13 reduced the levels of PKM1 polyubiquitination (Figure 4H; Figure S8D, Supporting Information). Moreover, through IP of truncated PKM1, we found that NUDT13 abolished the ubiquitin chains at the N‐terminus of PKM1 (Figure S8E, Supporting Information). These results indicate that NUDT13 stabilizes PKM1 by mitigating its ubiquitination.

We next investigated which types of ubiquitin chains on PKM1 were affected by NUDT13. Using a set of site‐directed mutants, we observed that both K48R and K63R ubiquitin mutants (unable to form K48 and K63 ubiquitin linkages, respectively) attenuated the impact of NUDT13 on PKM1 ubiquitination (Figure 4I; Figure S8F, Supporting Information). Accordingly, NUDT13 reduced K48‐ and K63‐polyubiquitination (able only to form K48 and K63 ubiquitin linkages, respectively), but did not affect K6‐polyubiquitination in both SW480 and DLD‐1 cells (Figure 4J; Figure S8G, Supporting Information). These results demonstrate that NUDT13 can counteract K48‐ and K63‐polyubiquitination at the N‐terminus of PKM1, thereby protecting PKM1 from degradation.

### 2.6 PKM1 ADP‐Ribosylation Catalyzed by PARP1 Leads to Its Ubiquitination and Degradation

**Figure 5:**
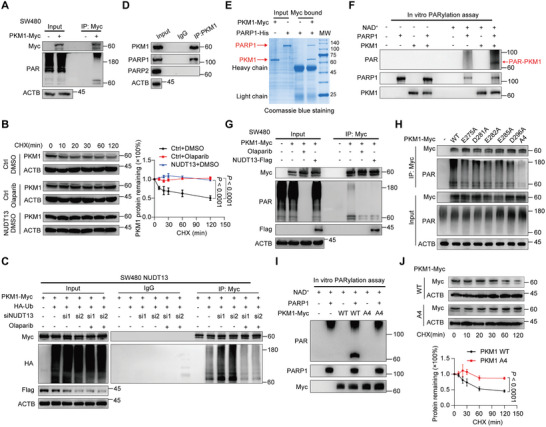
PARP1‐induced PKM1 ADP‐ribosylation signals for ubiquitination and degradation. A) In vivo PARylation assay was performed in SW480 cells transfected with PKM1‐Myc. B) Control and NUDT13‐overexpressing SW480 cells pre‐treated with DMSO or Olaparib (10 µm) were exposed to 50µg mL^−1^ CHX for the indicated time. Quantification of PKM1 by densitometry. C) Immunoblot analysis of PKM1 ubiquitination levels in NUDT13‐overexpressing SW480 cells transfected with NUDT13 siRNA, and treated with or without Olaparib (10 µm) for 48h. (D and E) The interaction between PKM1 and PARP1 was detected by Co‐IP D) and in vitro pull‐down assays E). F) In vitro PARylation assay to detect the PARylation of purified PKM1 catalyzed by recombinant human PARP1. G) Immunoblot analysis of PKM1 PARylation levels in SW480 cells treated with Olaparib (10 µm) for 48h, or transfected with NUDT13‐Flag plasmids. H) Immunoblot analysis of PKM1 PARylation levels in 293T cells transfected with WT or mutant PKM1. The amounts of PKM1 levels in different groups were adjusted by MG132 treatment. I) In vitro PARylation assay to detect the PARylation of purified PKM1. J) CHX chase assays were conducted to assess the stabilities of PKM1 WT and A4 mutant. All results mentioned above were obtained from 3 or more independent experiments. Data are presented as mean ± SD; *P* value was calculated by two‐way ANOVA (B and J).

Previous studies have reported that mouse NUDT13 possesses NAD^+^/NADH hydrolase activity.^[^ ^26^, ^27^ ^]^ Given that NAD^+^ serves as a substrate for ADP‐ribosylation, potentially priming ubiquitination,^[^ ^13^, ^28^ ^]^ we thus tested whether PKM1 ubiquitination is associated with its ADP‐ribosylation. In vivo poly ADP‐ribosylation (PARylation) assays showed that PKM1 was indeed PARylated (**Figure** **5**A; Figure S9A, Supporting Information). There are four ADP‐ribosyltransferases (PARP 1/2/5a/5b) that are primarily responsible for the synthesis of poly ADP‐ribose (PAR) chains.^[^ ^28^ ^]^ We set out to explore the potential ADP‐ribosyltransferases responsible for the PARylation of PKM1 and observed that treatment of CRC cells with Olaparib, a PARP 1/2 inhibitor, resulted in a significant upregulation of PKM1 levels (Figure S9B, Supporting Information) and enhanced its half‐life (Figure 5B), whereas XAV939, a PARP 5a/5b inhibitor, did not (Figure S9B, Supporting Information). We then investigated whether PKM1 PARylation mediates its ubiquitination and degradation. As shown in Figures 5C and S9C (Supporting Information), Olaparib completely abrogated the elevated PKM1 ubiquitination induced by NUDT13 silencing, suggesting that PKM1 PARylation governs its ubiquitination. Furthermore, we observed that PKM1 could directly bind to PARP1 but not PARP2 (Figure 5D,E; Figure S9D, Supporting Information), and the C‐terminus of PKM1 was imperative for the interaction (Figure S9E, Supporting Information). Additionally, PARP1 knockdown enhanced PKM1 protein levels, indicating that PKM1 PARylation is primarily catalyzed by PARP1 (Figure S9F, Supporting Information). In vitro PARylation assays confirmed that purified PKM1 could indeed be PARylated by PARP1 (Figure 5F). Moreover, Olaparib completely abrogated the PARylation of PKM1, and the overexpression of NUDT13 also reduced the PKM1 PAR chains (Figure 5G; Figure S9G, Supporting Information). These results identify PARP1 as the ADP‐ribosyltransferase responsible for PKM1 PARylation, a process that can be counteracted by NUDT13.

Utilizing truncation mutants of PKM1, we identified the 1–305 AA regions as the primary domain targeted for PARylation (Figure S9H, Supporting Information). To further delineate the PARylated sites within PKM1, we systematically mutated the potential residues predicted by ADPredict^[^ ^29^ ^]^(Figure S9I, Supporting Information). Our results indicated that residues E275/D281/E282/E285/D296 were susceptible to PARylation, and a multi‐point mutant (A4) greatly diminished the presence of PAR chains (Figure 5H,I; Figure S9J, Supporting Information). Additionally, PKM1‐A4 exhibited an extended half‐life compared to PKM1‐WT (Figure 5J), and PARP1 inhibition did not increase the levels of PKM1‐A4 (Figure S9K, Supporting Information), suggesting that these residues are major PARylation sites of PKM1. Moreover, mutation of these PARylated sites on PKM1 resulted in reduced ubiquitination but had no effects on its interaction with either NUDT13 or PARP1 (Figure S9L,M, Supporting Information).

In addition, we tested 8 PAR‐related E3 ligases using specific siRNAs,^[^ ^30^, ^31^, ^32^, ^33^, ^34^ ^]^ but none of them displayed a significant impact on PKM1 protein levels (Figure S9N, Supporting Information). Moreover, it has been shown that PAR polymers can mediate protein interactions,^[^ ^30^, ^31^ ^]^ but we found that Olaparib treatment did not affect the interaction between PKM1 and NUDT13 (Figure S9O, Supporting Information).

### 2.7 NUDT13 Suppress CRC Initiation by Directly Inhibiting PARP1‐Catalyzed PKM1 ADP‐Ribosylation

**Figure 6:**
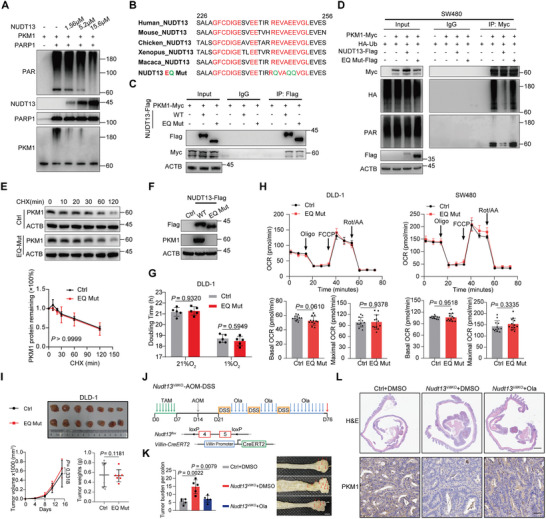
NUDT13 suppresses PKM1 ADP‐ribosylation to stabilize PKM1 protein. A) Immunoblot analysis of PKM1 PARylation levels after incubation of PARylated PKM1 with control or indicated concentrations of recombinant hNUDT13. B) Sequence alignment of the Nudix box motif of NUDT13 among different species. Highly conserved amino acids are shown in red. Mutant sites are shown in green. C) Co‐IP assay in 293T cells co‐transfected with PKM1‐Myc and NUDT13‐Flag WT or EQ mutant plasmids. D) Immunoblot analysis of PKM1 PARylation and ubiquitination levels in SW480 cells transfected with NUDT13‐Flag or EQ mutant plasmids. E) SW480 cells transfected with control or EQ mutant plasmids were exposed to 50µg/mL CHX for the indicated time. Quantification of PKM1 by densitometry. F) Immunoblot analysis of the PKM1 levels in DLD‐1 cells transfected with NUDT13‐Flag WT or EQ mutant plasmids. G) The doubling time of EQ mutant proficient DLD‐1 cells under normoxic or hypoxic conditions, as measured by CCK8. H) Top: the OCR of DLD‐1 and SW480 cells transfected with EQ mutant plasmids in response to oligomycin, FCCP, and rotenone/antimycin A. Bottom: bar graphs depicting the basal OCR (left) and the maximal OCR (right) of DLD‐1 and SW480 cells. I) Xenograft tumors formed in BALB/c nude mice (*n* = 7). Subcutaneous tumors were measured by volume and weight. J) Top: experimental scheme of Olaparib treatment schedule in the Nudt13*^VillKO^*‐AOM‐DSS mouse model. Olaparib was injected intraperitoneally every two days during the fresh drinking water period. Tumor burden was assessed at 55 days post‐AOM (red arrows). Bottom: diagram of the Nudt13*^flox^* and *Villin‐CreERT2* alleles. K) Macroscopic tumor burden formed in colons was counted in control and Nudt13*^flox^* mice, with or without Olaparib treatment (*n* = 5). Gross images of the distal colons are shown, and the red arrowhead indicates macroscopic tumors. Scale bars, 5 mm. L) Hematoxylin and eosin (H&E) and anti‐PKM1 staining of colon tumor sections from control and Nudt13*^flox^* mice, with or without Olaparib treatment. Scale bars, 2 mm or 50 µm. All results mentioned above were obtained from 3 or more independent experiments. Data are presented as mean ± SD; *P* values were calculated by two‐way ANOVA (E and I) and Student's *t‐*test (G, H, I, and K).

We next investigated the involvement of NUDT13's NADH pyrophosphatase activity in the regulation of PKM1. Detection of NAD^+^ levels in CRC cells revealed that NUDT13 could decrease both cellular NAD^+^ and NADH (Figure S10A, Supporting Information). However, replenishing NAD^+^ in NUDT13‐proficient cells (Figure S10B, Supporting Information) did not affect PKM1 protein levels but did enhance total cellular PARylation (Figure S10C,D, Supporting Information). To explore the involvement of common ADP‐ribosylation erasers in the regulation of the NUDT13‐PKM1 axis, we assessed the levels of PARylated PKM1 upon overexpression of PARG, ARH3, and TARG1.^[^ ^35^, ^36^ ^]^ Both NUDT13 and PARG could diminish the PARylation of PKM1, leading to an elevation in PKM1 levels (Figure S10E, Supporting Information). However, NUDT13 effectively diminish PARylated PKM1 even in the presence of PARGi (Figure S10F, Supporting Information), indicating that NUDT13 processes PKM1 independently of PARG. Consistently, silencing NUDT13 counteracted the impact of PARG on PKM1 (Figure S10G, Supporting Information). Previous reports have indicated the potential ADP‐ribose hydrolase activity of mouse NUDT13.^[^ ^26^, ^27^ ^]^ Therefore, we explored whether human NUDT13 inhibits PKM1 PARylation via its PAR processing activity. Incubation of eukaryotically purified NUDT13 with PARylated PKM1, revealed that NUDT13 could reduce the PAR linkage attached to PKM1 (**Figure** **6**A). To assess the specificity of NUDT13 in reducing PARylated PKM1, we examined its activity toward several well‐known PARylated proteins, including PARP1, histone H1, H2A, and H2B.^[^ ^37^, ^38^, ^39^ ^]^ As shown in Figure S10H–K (Supporting Information), NUDT13 exhibited little impact on the PARylation of these proteins, and we did not observe the interactions between NUDT13 and these proteins either (Figure S10L, Supporting Information). These results suggest that NUDT13 specifically diminishes the PARylation of PKM1 through its potential ADP‐ribose hydrolase activity, rather than its NADH pyrophosphatase activity or other ADP‐ribosylation erasers.

It has been reported that the glutamates within the Nudix box motif are crucial for the ADP‐ribose hydrolase activity of Nudix hydrolase.^[^ ^9^, ^13^ ^]^ Moreover, the sequence alignment of NUDT13 indicated that the glutamates within the Nudix box are conserved among different species (Figure 6B), suggesting an essential role for these glutamates in the biological function of NUDT13. Accordingly, we constructed a mutant vector of NUDT13 (E245/248/249Q), hereafter called the EQ mutant (Figure 6B). While the mutation did not disrupt the interaction between PKM1 and NUDT13 (Figure 6C), it failed to mitigate PKM1 PARylation and ubiquitination (Figure 6D; Figure S10M, Supporting Information), consequently leading to the inability to enhance PKM1 stability (Figure 6E) and protein levels (Figure 6F; Figure S10N, Supporting Information). Furthermore, the EQ mutant was unable to suppress the proliferation of CRC cells under hypoxia (Figure 6G; Figure S10O, Supporting Information), and abolished the effects of NUDT13 on glucose metabolism (Figure 6H; Figure S10P,Q, Supporting Information). Consistently, xenograft models demonstrated that the EQ mutant lost its ability to retard tumor growth (Figure 6I; Figure S10R, Supporting Information). These results indicate that the glutamates within the Nudix box motif are required for NUDT13 to decrease PKM1 PAR chains.

To substantiate the role of the NUDT13/PARP1‐PKM1 axis in CRC initiation, we employed a mouse model wherein we deleted Nudt13 selectively in the entire Villin+ intestinal epithelium (Nudt13*^VillKO^*). Tamoxifen (TAM) treatment effectively induced the generation of Nudt13*^VillKO^* mice (Figure S10S,T, Supporting Information). We found that Nudt13*^VillKO^* mice exhibited lower PKM1 protein levels in intestinal epithelia and developed more colon tumors in azoxymethane and DSS (AOM‐DSS)‐induced CRC models (Figure 6J–L; Figure S10T, Supporting Information). Interestingly, treatment with Olaparib reduced the tumor burden and elevated the PKM1 levels in tumor tissues (Figure 6K,L). Moreover, we assessed PKM1 and PARP1 levels in the same tissues as shown in Figure 1D. Consistently, PKM1 was downregulated while PARP1 was upregulated in intestinal carcinomas compared to polyps (Figure S10U, Supporting Information). These findings from two distinct mouse models of CRC further underscore the pivotal function of the NUDT13/PARP1‐PKM1 axis in CRC initiation.

### 2.8 The Residues 230–252 Within NUDT13 are Essential for Stabilizing PKM1 and Suppressing CRC Initiation

**Figure 7:**
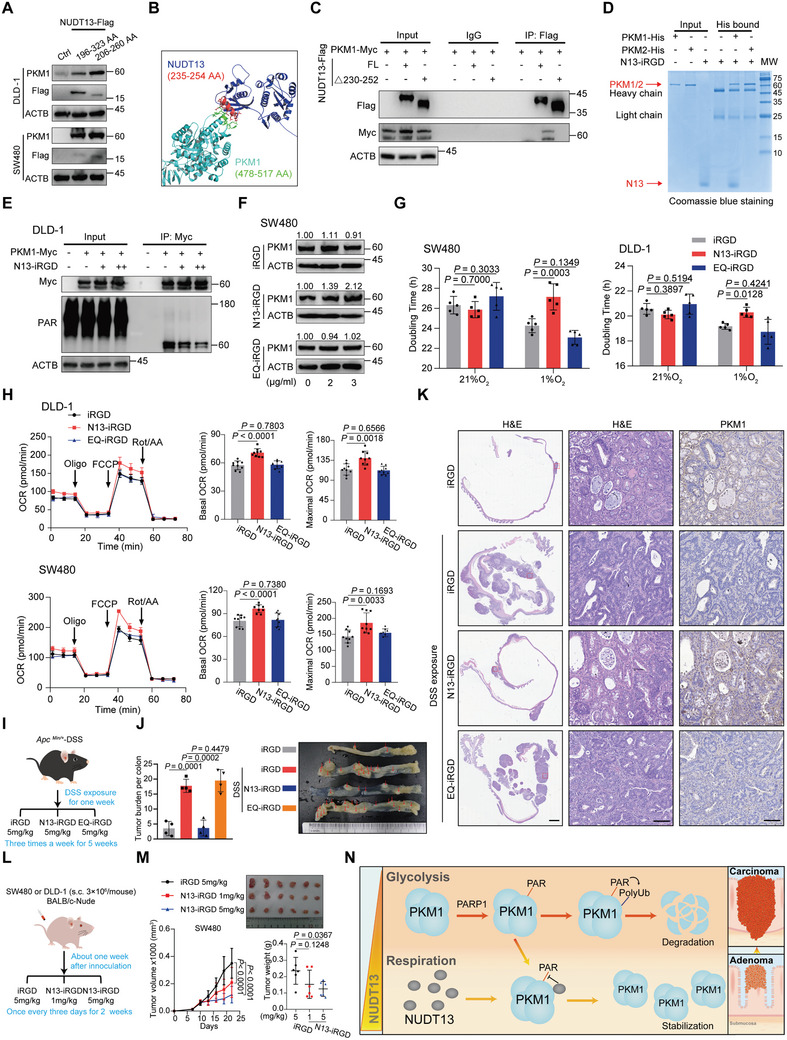
NUDT13 230–252 AA elicits tumor‐suppressive effects. A) Immunoblot analysis of PKM1 levels in DLD‐1 and SW480 cells transfected with NUDT13 truncated plasmids. B) Crystal structures of the predicted docking interfaces between PKM1 (green) and NUDT13 (red). C) Co‐IP assay in 293T cells co‐transfected with PKM1‐Myc and NUDT13‐Flag WT, or △230–252 plasmids. D) Coomassie blue staining showed the direct interaction between N13 peptide and purified PKM1, but not N13 peptide and PKM2, by in vitro pull‐down assay. E) Immunoblot analysis of PKM1 PARylation levels in DLD‐1 cells treated with different doses of N13‐iRGD for 48 h. F) Immunoblot analysis of PKM1 protein levels in SW480 cells treated with the indicated doses of fusion peptides for 48h. G) The doubling time of SW480 and DLD‐1 cells treated with indicated peptides under normoxic or hypoxic conditions, as measured by CCK8. H) The OCR of DLD‐1 and SW480 cells treated with indicated peptides in response to oligomycin, FCCP, and rotenone/antimycin A. I) Experimental scheme of the peptide treatment in *Apc^Min/+^*‐DSS mouse model. J) Macroscopic tumor burden formed in colons was counted (*n* = 4). The red arrowhead indicates macroscopic tumors. K) H&E and anti‐PKM1 staining of colon tumor sections from mice treated with control peptide, N13‐iRGD, or EQ‐iRGD. Scale bars, 2 mm or 100 µm. L) Experimental scheme of the peptide treatment in subcutaneous xenograft mouse model. M) Xenograft tumors formed in BALB/c nude mice (*n* = 6). Subcutaneous tumors were measured by volume (left) and weight (right). N) Schematic model illustrating the role of the NUDT13‐PKM1 axis in the regulation of CRC initiation. All results mentioned above were obtained from 3 or more independent experiments. Data are presented as mean ± SD; *P* values were calculated by Student's *t‐*test (G, H, J, and M) and two‐way ANOVA (M).

To determine the exact region of NUDT13 responsible for PKM1 regulation, we further constructed three truncated vectors of its NH domain (Figure S11A, Supporting Information). Compared to the other two vectors, the residues 206–260 held the strongest binding capacity for PKM1 (Figure S11B, Supporting Information), and could significantly upregulate PKM1 protein levels (**Figure** **7**A). Meanwhile, we conducted a docking simulation of NUDT13 and PKM1, and scrutinized the NUDT13‐PKM1 docking interface using PISA in PDBe.^[^ ^40^ ^]^ The predicted 193–274 and 235–254 AA regions of NUDT13 were identified as the potential docking moieties (Figure 7B; Figure S11C, Supporting Information), which were consistent with our IP results mentioned above (Figure S11B, Supporting Information). Interestingly, the predicted 235–254 AA region overlaps with the conserved Nudix box motif (230–252 AA), suggesting that the Nudix box might be required for both protein interaction and reducing ADP‐ribosylation. Indeed, deletion of the NUDT13 230–252 AA abolished the interaction between NUDT13 and PKM1 (Figure 7C). Taken together, these results indicate that 230–252 AA of NUDT13 is sufficient for binding and stabilizing PKM1.

We next synthesized a mimic peptide of NUDT13 230–252 AA conjugated with the membrane‐penetrating peptide iRGD (N13‐iRGD), and assessed whether this peptide can emulate the function of NUDT13 in regulating PKM1. Pull‐down assays confirmed that the N13 peptide could directly interact with PKM1 but not with PKM2 (Figure 7D). Subsequent treatment of CRC cells with N13‐iRGD resulted in a dose‐dependent reduction of PKM1 PARylation and an increase in PKM1 levels in both DLD‐1 and SW480 cells, while treatment with EQ‐iRGD (E245/248/249Q in N13‐iRGD) failed to affect PKM1 PARylation and protein levels (Figure 7E,F; Figure S11D–F, Supporting Information). Moreover, we investigated whether N13‐iRGD can phenocopy the effects of NUDT13 on glycometabolic reprogramming. Notably, N13‐iRGD, but not EQ‐iRGD, led to a prolonged doubling time under hypoxia, enhanced OXPHOS, and reduced glycolysis in CRC cells (Figure 7G,H; Figure S11G,H, Supporting Information).

Next, we examine the impact of N13‐iRGD on CRC initiation in *Apc ^Min/+^*‐DSS mouse models (Figure 7I). Strikingly, colons from mice treated with N13‐iRGD presented significantly fewer tumors, reduced histologic dysplasia, and higher PKM1 levels, comparable to mice without DSS exposure (Figure 7J,K). In contrast, EQ‐iRGD treatment did not yield an appreciable effect. In subcutaneous xenograft models, N13‐iRGD resulted in markedly lower tumor volumes and weights (Figure 7L,M; Figure S11I, Supporting Information). Furthermore, in mice treated with these peptides, no observable abnormal symptoms or histological abnormalities in vital organs were detected, indicating their tolerability (Figure S11J–M, Supporting Information). These findings identify 230–252 AA of NUDT13 as the vital moiety for modulation of PKM1, which could serve as a promising target for developing drugs to suppress CRC initiation and progression.

Given the conservation of the Nudix box in the NUDT family, we wondered whether other members of the NUDT family have similar effects on PKM1. We constructed another 21 NUDT family vectors and found that only NUDT13 could upregulate PKM1, suggesting that NUDT13‐mediated regulation of PKM1 is somewhat specific (Figure S11N, Supporting Information).

## 3 Discussion

The majority of CRCs follow an adenoma‐carcinoma multistep sequence, characterized by the gradual accumulation of genomic alterations.^[^ ^2^ ^]^ Therefore, deciphering the alterations between adenomatous polyps and CRC may be more relevant than comparing normal tissues with established CRC. Through analysis of the transcriptome profiles extracted from CRC tissues, adenomatous polyps, and para‐tumor tissues, we identified NUDT13 as a hitherto underestimated regulator that can impede malignant colorectal transformation. Our results showed that NUDT13 enhances OCR while decreasing lactate production, thus inducing an OXPHOS phenotype in CRC cells and retarding cell expansion under hypoxia. Notably, the PARP1 inhibitor Olaparib significantly reduces tumor burden in Nudt13*^VillKO^* mice, implying that CRC patients with low NUDT13 protein levels may benefit from Olaparib treatment. Additionally, our clinical sample analysis revealed a negative correlation between NUDT13 levels and clinical stage, suggesting that NUDT13 may influence various stages of CRC tumorigenesis. These findings underscore the modulatory role of NUDT13 in metabolic remodeling during CRC initiation and the potential clinical significance of targeting NUDT13.

PKM catalyzes the last committed step of glycolysis, converting phosphoenolpyruvate to pyruvate.^[^ ^41^ ^]^ Two PKM isoforms, which are splice variants with different activities, influence the pathway of glucose metabolism in tumor cells.^[^ ^15^, ^17^, ^18^, ^42^ ^]^ PKM1 promotes glucose oxidation, while PKM2 generally signals for fermentation.^[^ ^15^, ^16^, ^17^, ^18^, ^24^, ^43^ ^]^ Given that aerobic glycolysis is known for its ability to provide conditions catering to the needs of proliferative tumor cells, PKM1 may inhibit tumorigenesis by disrupting glycolysis. Moreover, emerging evidence suggests that switching the PKM isoform to PKM1 markedly hinders tumor progression in various cancers.^[^ ^15^, ^18^, ^44^ ^]^ Here, we identified PKM1 as a direct substrate of NUDT13 and PARP1, and that PKM1 is responsible for the OXPHOS phenotype and tumor‐suppressive effect induced by NUDT13. However, we cannot rule out the potential involvement of non‐cell‐autonomous mechanisms, probably mediated by stromal cells within the TME. This intriguing question warrants further investigation. Additionally, prior research has proposed an aggressive role of PKM1 in small cell lung cancer (SCLC).^[^ ^45^ ^]^ Hence, we speculate that the expression pattern and biological function of PKM in tumors are tissue‐specific and context‐dependent.^[^ ^41^, ^46^ ^]^

ADP‐ribosylation is a well‐studied post‐translational modification involving the covalent attachment of one or more ADP‐ribose units to target proteins by ADP‐ribosyltransferases, such as PARPs and Sirtuins.^[^ ^28^ ^]^ In mammals, PARylated proteins regulate a wide range of biological processes, including DNA damage repair, signal transduction, and chromatin remodeling.^[^ ^28^ ^]^ Recently, ADP‐ribosylation has emerged as an important signal for protein ubiquitination and degradation.^[^ ^30^, ^32^, ^33^ ^]^ In this study, we confirmed that PKM1 can be PARylated by PARP1 at E275/D281/E282/E285/D296 and subsequently degraded through K48‐ and K63‐ubiquitination. However, despite screening several well‐characterized PAR‐related E3 ubiquitin ligases, we were unable to identify the specific E3 ligase responsible for this process in the context of PKM1. This suggests that the crosstalk between PKM1 PARylation and ubiquitination may involve a distinct, as‐yet‐unidentified E3 ligase. Notably, ADP‐ribosylation is a reversible modification that can be removed by various hydrolases, such as PARG, ARH3, and Nudix hydrolases.^[^ ^28^ ^]^ NUDT16 has been implicated in the response to DNA damage repair by removing the ADP‐ribosylation of 53BP1, CtIP, and SETD3,^[^ ^13^, ^47^, ^48^ ^]^ while NUDT5 and NUDT9 are known to play important roles in energy metabolism by processing ADP‐ribose.^[^ ^27^ ^]^ These studies suggest that the NUDT family is closely related to DNA damage and metabolism. Given NUDT13's predominant chromatin‐bound sublocation, it is plausible that NUDT13 plays a role in DNA damage repair by regulating protein PARylation. Moreover, multiple studies have shown that PARylation mediates the crosstalk between DNA damage and glycometabolism.^[^ ^49^, ^50^ ^]^ Here, we found that NUDT13 collaborates with PARP1 to modulate the PARylation of PKM1, enhancing our understanding of the role of PARylation in glycometabolism. Specifically, NUDT13 prevents the PARylation of PKM1 through its potential ADP‐ribosylation hydrolase activity, rather than its conventional NADH pyrophosphatase activity. Furthermore, we validated the crosstalk between NUDT13, PKM1, and PARP1 using two distinct mouse models of intestinal tumorigenesis, underscoring their conserved and critical roles in CRC initiation. More interestingly, we found that a conserved stretch of NUDT13 (residues 230–252) is necessary and sufficient for mitigating PKM1 PARylation, offering potential for the development of cancer therapeutic strategies.

In conclusion, we identified NUDT13 as a roadblock in the progression from colorectal adenoma to CRC, and revealed that NUDT13 stabilizes PKM1 protein by reducing the PARylation that is catalyzed by PARP1, thereby reprogramming the metabolic propensity of CRC cells (Figure 7N). Inspiringly, a recombinant peptide derived from the most conserved stretch of NUDT13 exhibits a similar suppressive effect on CRC. Although further development would be needed to optimize the delivery system, our findings lay the groundwork for innovative clinical remedies in the future.

## 4 Experimental Section

### Ethics Approval

The research complies with all related ethical regulations of Sun Yat‐sen University Cancer Center. All animal protocols were approved by the Sun Yat‐sen University Animal Care and Use Committee (L102012017008V) and were performed following the guidelines. The use of clinical samples was approved by the Institutional Review Board of Sun Yat‐sen University Cancer Center (GZR2020‐219) and all clinical materials were obtained from the Department of Pathology and Department of Endoscopy at Sun Yat‐sen University Cancer Center.

### Animal Study

All animals were housed in individually ventilated cages at the Center of Experimental Animal of SYSUCC, with suitable temperature and humidity conditions, as well as sustained access to food and water. The study was approved by the Sun Yat‐sen University Animal Care and Use Committee. Animals were randomized using a computer based random order generator.

For subcutaneous xenograft model, NUDT13‐overexpressing DLD‐1, SW480 (3 × 10^6^), or NUDT13 knockout DLD‐1 and SW480 cells (1.5 × 10^6^) resuspended in FBS‐free medium were subcutaneously injected into the flanks of 4‐week‐old nude mice (Vital River Laboratories, Beijing, China, RRID: IMSR_RJ: BALB‐C‐NUDE). Tumor growth was monitored every 3 days, and mice were sacrificed after 2 or 3 weeks. The tumors were then harvested, weighed, fixed, and paraffin‐embedded for further analysis.

For the *Apc ^Min/+^*‐DSS‐induced mouse CRC model, 8‐week‐old *Apc ^Min/+^* mice (Gempharmatech, Jiangsu, China, RRID: IMSR_GPT: T001457) were fed with 2%DSS (0216011080, MP, Canada) water for 1 week and sacrificed after 5 weeks. For the Nudt13*^VillKO^*‐AOM‐DSS model, Nudt13*^fl/fl:Vill‐creER^* mice were generated by crossing Nudt13 floxed mice (Cyagen Biosciences, USA, RRID: IMSR_GPT: T040575) with *Villin*‐*CreERT2* (RRID: IMSR_JAX: 020282) mice. 8 to 10‐week‐old Nudt13 *^fl/fl:Vill‐creER^* mice were subjected to 7 consecutive days of tamoxifen (10540‐29‐1, Sigma–Aldrich, USA) injection (100 µL 20 mg·mL^−1^) to generate Nudt13*^VillKO^* mice. 7 days after tamoxifen injection, mice were injected intraperitoneally with 10 mg·kg^−1^ AOM (25843–45‐2, Sigma–Aldrich, USA). After 7 days, the mice were fed with 2% DSS water for 5 days followed by 10 days of regular drinking water. Then, repeated DSS induction for 2 rounds and the mice were sacrificed on the 76th day. Colon tissues were collected for further analysis.

### Seahorse Analysis

The oxygen consumption rate (OCR) and extracellular acidification rate (ECAR) were measured with Seahorse XFe96 Analyzer (Agilent, USA, RRID: SCR_019545) by Seahorse XF Cell Mito Stress Test Kit (103015–100, Seahorse, USA) and Seahorse XF Glycolysis Stress Test Kit (103020–100, Seahorse, USA), respectively. Briefly, 15000 DLD‐1 or 20 000 SW480 cells were seeded in 96‐well Seahorse XFe96 cell culture plates and cultured with 10%FBS RPMI 1640 overnight. Cells were washed twice and incubated with Seahorse XF RPMI Medium (103576–100, Seahorse, USA) supplemented with 2 mm glutamine (103579–100, Seahorse, USA), 10 mm glucose (103577–100, Seahorse, USA), and 1 mm pyruvate (103578–100, Seahorse, USA) (OCR) or with 2 mm glutamine only (ECAR) at 37 °C for 45–60 min in a CO_2_‐free incubator. OCR was measured by adding 1.5 mm oligomycin, 0.5 mm FCCP, and 0.5 mm rotenone/antimycin A. ECAR was measured by adding 10 mm glucose, 1 mm oligomycin, and 50 mm 2‐DG. Results were normalized by protein concentration and displayed in pmol·min^−1^ (OCR) and mpH·min^−1^ (ECAR). Three experimental replicates were performed.

### In Vitro PARylation and De‐PARylation Assays

The purified recombinant PKM1, H1, H2A, or H2B (1 µg) was mixed with 1 µg recombinant human PARP1 (Sino Biological) with or without NAD^+^ (HY‐B0445, MCE, China) in 1×reaction buffer (50 mm Tris‐HCl (pH 8.0), 4 mm MgCl_2_, 2 0mm NaCl, 1 mm DTT, and 100 ng sheared DNA (D7656, Sigma–Aldrich, USA)) at 37 °C for 30 min. The reactions were stopped by the addition of 5 × SDS loading buffer. PARylation of indicated proteins were analyzed by WB using anti‐PAR antibody.

For the de‐PARylation assay, in vitro PARylation reactions were stopped by the addition of 1 µm Olaparib (S1060, Selleck, USA), then added different concentrations of purified NUDT13 and adjusted the Mg^2+^ concentration to 15 mm by MgCl_2_. Incubated the reaction mixture for 2 h at 37 °C and stopped the reaction by the addition of 5 × SDS loading buffer.

### Molecular Docking Simulation

The 3D structure of NUDT13 was predicted by AlphaFold (RRID: SCR_025454), and the 3D structure of PKM1 (PDB: 3SRF) was obtained from the Swiss‐Model (RRID: SCR_018123). Protein‐protein docking simulation was performed in the GRAMM‐X program, and the results were visualized and analyzed in PISA (Proteins, Interfaces, Structures, and Assemblies, RRID: SCR_015749). Molecular graphics were generated by PyMOL (RRID: SCR_000305).

### Statistical Analysis

All statistical analyses were conducted using GraphPad Prism (Version 8; La Jolla, CA, USA, RRID: RRID: SCR_002798) or SPSS (Version 25.0; Armonk, NY, USA, RRID: SCR_016479). The associations of NUDT13 or PKM1 levels with the patient's clinicopathologic parameters were analyzed by the Pearson chi‐square test. Univariate or multivariate survival analyses were assessed using the COX proportional hazards regression model; The correlation between NUDT13 and PKM1 was performed by Pearson correlation analysis. For the comparisons between the two groups, two‐tailed Student's *t‐*tests were used. ANOVA was used for multiple comparisons among more than two groups. The data were shown as mean ± SD, and *P* < 0.05 was considered statistically significant.

## Conflict of interest

The authors declare no conflicts of interest.

## Author Contributors

F.‐W.W. conceived and devised the study. J.‐L.L. and F.‐W.W. designed the experiments and analysis. Y.‐X.Y., J.‐H.C. and K.H. provided constructive guidance and advice. J.‐L.L., Y.‐X.Y., B.‐X.Z., J.‐H.C., Y.‐R.L., S.‐D.X., S.‐Y.L. and C.‐J.H. performed biological experiments. J.‐L.L. and J.‐H.C. performed animal experiments. J.‐L.L., Y.‐X.Y., B.‐X.Z., and J.‐W.C. analyzed the data. J.‐W.C., K.H., and Y.‐F.C. provided CRC patient tissue samples and clinical information. D.X. and F.‐W.W. supervised the research. J.‐L.L. and F.‐W.W. wrote the paper. All of the authors approved the submitted manuscript.

## Supporting information



Supporting Information

Supporting Information

Supporting Information

## Data Availability

RNA‐seq data extracted from CRCs, para‐tumor normal tissues, and adenomas have been deposited in the Gene Expression Omnibus under accession number: GSE228012 (corresponding SRA accession number: PRJNA940523). LC‐MS/MS raw data are available at the iProX with the identifier PXD041430.
