# Supplementary material for: Nudix Hydrolase 13 Impairs the Initiation of Colorectal Cancer by Inhibiting PKM1 ADP‐Ribosylation
Source: Adv Sci (Weinh). 2025 Feb 8;12(13):2410058. doi: 10.1002/advs.202410058 (PMC11967829; doi:10.1002/advs.202410058)
Supplement: Supplementary file 1 — Supporting Information [file ADVS-12-2410058-s001.docx]

**Supporting Methods and Materials for**

**Nudix Hydrolase 13 Impairs the Initiation of Colorectal Cancer by Inhibiting PKM1 ADP-Ribosylation**

Jinlong Lin, Yixin Yin, Jinghua Cao, Bingxu Zou, Kai Han, Yufan Chen, Siyu Li, Cijun Huang, Jiewei Chen, Yongrui Lv, Shuidan Xu, Dan Xie, Fengwei Wang

**Cell culture**

All cell lines were obtained from American Type Culture Collection (ATCC) and maintained in a 5% CO_2_ incubator (Thermo Fisher Scientific, Waltham, MA, USA) at 37°C with humidified sterile water. The CRC cell lines SW480 (RRID: CVCL_0546) and DLD-1(RRID: CVCL_0248) were cultured in Roswell Park Memorial Institute 1640 (RPMI 1640) medium (C11875500BT, Gibco, USA) supplemented with 10% fetal bovine serum (FBS) (10099141, Gibco, USA). HEK293T (RRID: CVCL_0063) and Hela (RRID: CVCL_0030) cells were cultured in Dulbecco’s modified Eagle’s medium (DMEM) (C11995500BT, Gibco, USA) supplemented with 10% FBS. Both RPMI 1640 and DMEM media were routinely supplemented with Penicillin-Streptomycin Solution (BL505A, Biosharp, China). Resuscitated cells were not cultured for more than 2 months *in vitro* to ensure optimal cell viability and growth.

**Patients and samples**

A total of 174 cases of primary colorectal cancer (CRC) tissues and corresponding para-cancerous normal tissues collected from patients who underwent surgical resection between March 2006 and October 2011 at the Sun Yat-sen University Cancer Center (SYSUCC) in Guangzhou, China were used for the analysis of clinicopathological features of NUDT13 and PKM1. Another cohort of 36 paired early-stage CRC tissues and 14 paired adenomas were collected between January and June in 2016 and between January and February in 2024, respectively, at the SYSUCC, for RNA extraction and RT-qPCR analysis. The correlation between NUDT13 and PKM1 was examined in the 174 CRC samples mentioned above. All cases had clear pathological diagnoses, and had not received any local or systemic treatment prior to tissue collection. Tumor staging and grading were determined according to the criteria established by the World Health Organization (WHO) and the sixth edition of the TNM classification of the International Union Against Cancer (UICC, 2009). The Institutional Review Board of Sun Yat-sen University Cancer Center approved this study.

**Western Blotting (WB)`**

IP lysis buffer (50 mM pH 7.4 Tris-HCl, 150 mM NaCl, 1 mM EDTA, 0.5% NP-40, and 10% glycerol) supplemented with protease inhibitor cocktail (CW2200S, CWBIO, China) was used to lyse cells on ice for 30 min. The supernatant was collected after centrifugation, then determined the protein concentration by the bicinchoninic acid (BCA) assay kit (23227, Invitrogen, USA). Denatured proteins were separated by 7.5-15% SDS-PAGE gels and then transferred to a polyvinylidene fluoride (PVDF) membrane (3010040001, Roche, Switzerland). The membranes were incubated with the respective antibody at 4 ℃ overnight after blocking, then visualized by ChemiDoc Touch (Bio-Rad, USA, RRID:SCR_021693) or by e-Blot Touch Imager (e-Blot, Shanghai, China). The antibodies used in this study are listed in Supplementary Table S5.

**RT-qPCR**

Total RNA was extracted from indicated cells or tissues using TRIzol (15596018, Invitrogen, USA). 1 μg of RNA was used to obtain cDNA by reverse transcription using PrimeScript™ RT reagent Kit (RR036A, TAKARA, Japan). According to the manufacturer’s instructions, qPCR was performed using 2×Color SYBR Green qPCR Master Mix (ROX2 plus) (A0012-R2, EZBioscience, China). The primer sequences are listed in Supplementary Table S6.

**siRNA and plasmids**

siRNAs used in this study were synthesized by RiboBio (Guangzhou, China). Detailed oligonucleotide sequences of siRNA are listed in Supplementary Table S7.

The short hairpin RNAs (shRNAs) in the pLKO vector specifically against PKM1 or NUDT13 were synthesized by Tsingke (Guangzhou, China). The sequences of shRNA used to target PKM1 or NUDT13 are listed in Supplementary Table S7.

The guide RNA (gRNA) specifically against NUDT13 was cloned into the LentiCRISPR-V2 vector. The sequence is listed in Supplementary Table S7.

Wild type (WT) and mutants of NUDT13 were subcloned into the plvx-puro lentivirus vector. WT and truncated mutants of PKM1 were subcloned into the pcDNA3.1 vector. WT and mutants of Ub were kindly provided by Professor Xiao-feng Zhu (Sun Yat-sen University Cancer Center).

**Immunoprecipitation (IP)**

Equal cell lysates were incubated with protein A/G magnetic beads (HY-K0202, MCE, China) at 4℃ for 1h to pre-clear the nonspecific proteins. Then discarded the beads and added the pre-washed new protein A/G magnetic beads mixed with specific antibodies, or added pre-coated magnetic beads (anti-Flag (B26102), anti-Myc (B26302), or anti-HA (B26201), Bimake, USA), and incubated by a rotator at 4 ℃ overnight. Washed the beads using pre-cold IP lysis buffer for 10min at 4℃ and repeated 4 times. Discarded the IP lysis buffer and denatured the samples in 1×SDS buffer for further WB analysis.

**RNA-seq analysis**

Total RNA was extracted from patient samples using TRIzol (15596018, Invitrogen, USA). RNA-seq analysis was performed by BGI (Shenzhen, China, RRID: SCR_011114) using the MGISEQ-2000 platform. The raw data filtered by SOAPnuke (v1.5.6, RRID: SCR_015025) were mapped to the hg19 genome with HISAT (v2.1.0, RRID: SCR_015530). RSEM (v1.3.1, RRID: SCR_000262), pheatmap (v1.0.8, RRID:SCR_016418), and DESeq2 (v1.4.5, RRID:SCR_015687) were used to analyze RNA-seq data according to the manufacturer’s instructions.

***In vivo* ubiquitination assays**

SW480, DLD-1, or 293T cells transfected with HA-Ub and other plasmids were incubated with 10 μM MG132 for 6h before lysis. Lysate concentrations were estimated using a BCA kit and equal lysates were then subjected to IP with indicated beads, followed by immunoblot with the antibodies as indicated in the figures.

**Immunohistochemistry (IHC)**

The paraffin tissue sections were first baked at 56℃ for 30 min and then subjected to dewaxing in dimethylbenzene and rehydration with graded ethanol. Antigen retrieval of the sections was performed using high pressure and heat repair. After natural cooling, the sections were incubated with 3% hydrogen peroxide for 10 min to inactivate the endogenous peroxidase. Subsequently, the sections were blocked with 1% BSA at 37℃ for 1h and incubated with anti-NUDT13 antibody at a 1:50 dilution or anti-PKM1 antibody at a 1:100 dilution at 4℃ overnight. A secondary antibody (PV6000, Zsbio, China) was used to bind the primary antibody at 37℃ for 1h, and DAB (ZLI9017, Zsbio, China) was used to stain the target protein, followed by hematoxylin staining.

***In vitro* proliferation and cytotoxicity experiments**

Cell proliferation was measured using the Cell Counting Kit 8 (CCK8) (HY-K0301, MCE, China) and colony formation assays. For CCK8 assays, 3000 CRC cells were seeded in 3-5 replicates in a 96-well plate. The medium was replaced with FBS-free RPMI 1640 containing CCK8 (90 μL RPMI 1640 and 10 μL CCK8 for each well). Absorbance was measured after 2h at 450 nm using VersaMax (MD, USA).
 For the colony formation assays, 300 cells were seeded in 3 replicates in a 12-well plate. Visible colonies were fixed with paraformaldehyde after 7-10 days, and the colonies were stained with crystal violet.
 For hypoxic culture, 3000 cells were seeded in 3-5 replicates in a 96-well plate. The 96-well plate was then placed in an anaerobic jar and the air was adjusted to 1% O_2_ using Anoxomat Mark II (Anoxomat, Netherlands). After 2 days, cell proliferation was measured using CCK8, and the absorbance was converted to the cell number using a previously constructed growth curve.
 For cell drug susceptibility assays, cells were seeded in a 96-well plate or 12-well plate on the first day. The next day, the medium was replaced with RPMI 1640 containing Oligomycin A (S1478, Selleck, USA), rotenone (HY-B1756, MCE, China), gboxin (S8828, Selleck, USA), or teriflunomide (S4169, Selleck, USA). Further analysis was conducted using CCK8 or colony formation assays.
**Subcellular Protein Fractionation**

Separation and preparation of cytoplasmic, nuclear soluble, and chromatin-bound protein from cultured cells was completed by the Subcellular Protein Fractionation Kit (78840, Thermo Scientific, USA). In brief, added the cytoplasmic extraction buffer to the cell pellet and centrifuged at 500×g for 5 min. Transferred the supernatant (cytoplasmic extract) and added nuclear extraction buffer to the pellet, incubated on ice for 30 min with gentle mixing, and then centrifuged at 5000×g for 5 min. Transferred the supernatant (soluble nuclear extract) and added room temperature nuclear extraction buffer containing CaCl_2_ and Micrococcal Nuclease to the pellet. Incubated in the water bath at 37℃ for 5 min and centrifuged the tube at 16000×g for 5 min, the supernatant was chromatin-bound nuclear extract. Transferred the supernatant and added room temperature pellet extraction buffer containing protease inhibitors, vortex on the highest setting for 15 seconds, and centrifuged at 16000×g for 5 min after incubating at room temperature for 10 min, the supernatant was the cytoskeletal extract.

**NAD^+^/NADH detection assay**

Cellular NAD^+^ and NADH were detected by CheKine Micro Coenzyme I NAD(H) Assay Kit (KTB1020, Abbkine, China) according to the instruction of the manufacturer. In brief, acidic and alkaline extraction buffers were used to extract NAD^+^ and NADH, respectively. Then the samples were heated in a water bath at 60℃ for 5 min and neutralized. After an enzyme-based reaction, the chromogenic reaction was induced by adding WST-8. Measured the absorbance after 30min at 450nm by VersaMax (MD, USA) and normalized by protein concentration.

**Pull-down assay and** **Coomassie blue staining**

For pull-down assays between NUDT13 and PKM1, or PKM1 and PARP1, 20 μg of purified recombinant PKM1-his, NUDT13-Flag, PKM1-Myc, or control peptides were incubated with 20 μL pre-washed anti-His (HY-K0209, MCE, China), anti-Flag, or anti-Myc coated beads in IP lysis buffer at 4 ℃ for 4h. Washed the beads five times with IP lysis buffer and incubated with 20 μg of another recombinant protein at 4 ℃ overnight. The beads were washed five times the next day and then denatured in 1×SDS for further SDS-PAGE analyses. After electrophoresis, the gels were washed in double distilled water and then stained with Coomassie blue (PT0018, Leagene, Beijing) for at least 4h. The non-specific staining was destained with elution buffer (10% acetic acid, 40% methanol, and 50% double distilled water) overnight.

For pull-down assays between PKM1/2 and N13 peptide (Genscript, China), 20 μg of the purified recombinant PKM1-His, PKM2-His, or 6×His peptide was incubated with 20 μL pre-washed anti-His coated beads in IP lysis buffer at 4 ℃ for 4h. Washed the beads five times with IP lysis buffer and incubated with 20 μg of N13 at 4 ℃ overnight. The remaining steps are the same as before.

**Silver staining and LC-MS/MS analysis**

After electrophoresis, the gels were washed with double distilled water. Protein gels were stained using the Fast Silver Stain Kit (P0017S, Beyotime, China) according to the manufacturer's instructions. The different strips were then excised and subjected to in-gel digestion. Further LC-MS/MS analysis was performed by Fitgene (Guangzhou, China). Protein identification was carried out using Mascot (RRID: SCR_014322), based on the Uniprot database (RRID: SCR_002380). Proteins identified from the immunoprecipitants pulled down with NUDT13 are listed in Supplementary Table S8.

**Protein expression and purification**

Human PKM1, PKM2, and PARG were cloned into a pET-28a vector, and the recombinant vector was expressed in *Escherichia coli* BL21 (DE3) in Luria-Bertani medium containing 0.1mg·mL^-1^ ampicillin and 34 μg·mL^-1^ chloromycetin. After induction with 0.5 mM IPTG when the optical density at 600 nm was 0.6-0.8, cells were cultured at 15 ℃ overnight. Then cells were collected and lysed with lysis buffer (10 mM imidazole, 50 mM NaH_2_PO_4_, 1 mM PMSF, 300 mM NaCl, pH 8.0), and centrifuged at 18000 rpm for 1h. Pre-equilibrated the Ni-NTA column with lysis buffer and loaded the cell supernatant into the column after being filtered by 0.45 μm strainer. Washed the column with wash buffer (lysis buffer with 30 mM imidazole) and then eluted the purified proteins with elution buffer (lysis buffer with 300 mM imidazole). The purified proteins were further dried by ALPHA1-2LD plus (Christ, Germany) and dissolved with PBS. SDS-PAGE was used to analyze the target protein.

Human recombinant NUDT13 protein was purified by Abmart (Shanghai, China) from HEK293T cells. Human recombinant histone H1, H2A, and H2B proteins were purified by Active Motif (USA) from *E. coli*. All the peptides used in this study were synthesized by Genscript (Nanjing, China).

**Supporting Figures for**

**Nudix Hydrolase 13 Impairs the Initiation of Colorectal Cancer by Inhibiting PKM1 ADP-Ribosylation**

Jinlong Lin, Yixin Yin, Jinghua Cao, Bingxu Zou, Kai Han, Yufan Chen, Siyu Li, Cijun Huang, Jiewei Chen, Yongrui Lv, Shuidan Xu, Dan Xie, Fengwei Wang

**Figure S1**

**
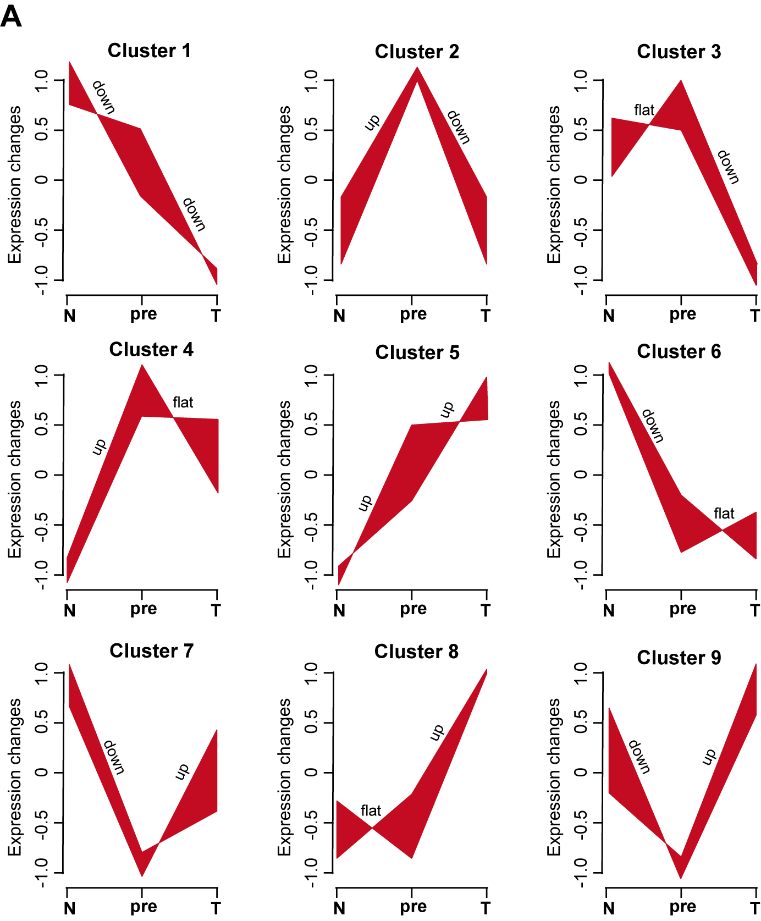
**

**Figure S1. Identification of the driver genes regulating CRC initiation via gene expression pattern analysis. (A)** Clustering analysis of the RNA-seq among the early-stage CRC tissues, the precancerous lesions, and normal samples. T: tumor. Pre: the precancerous lesions. N: normal. Gene expression patterns in cluster 7 and cluster 9 are as follows: cluster 7 (N>T>pre), cluster 9 (T>N>pre).

**Figure S2**


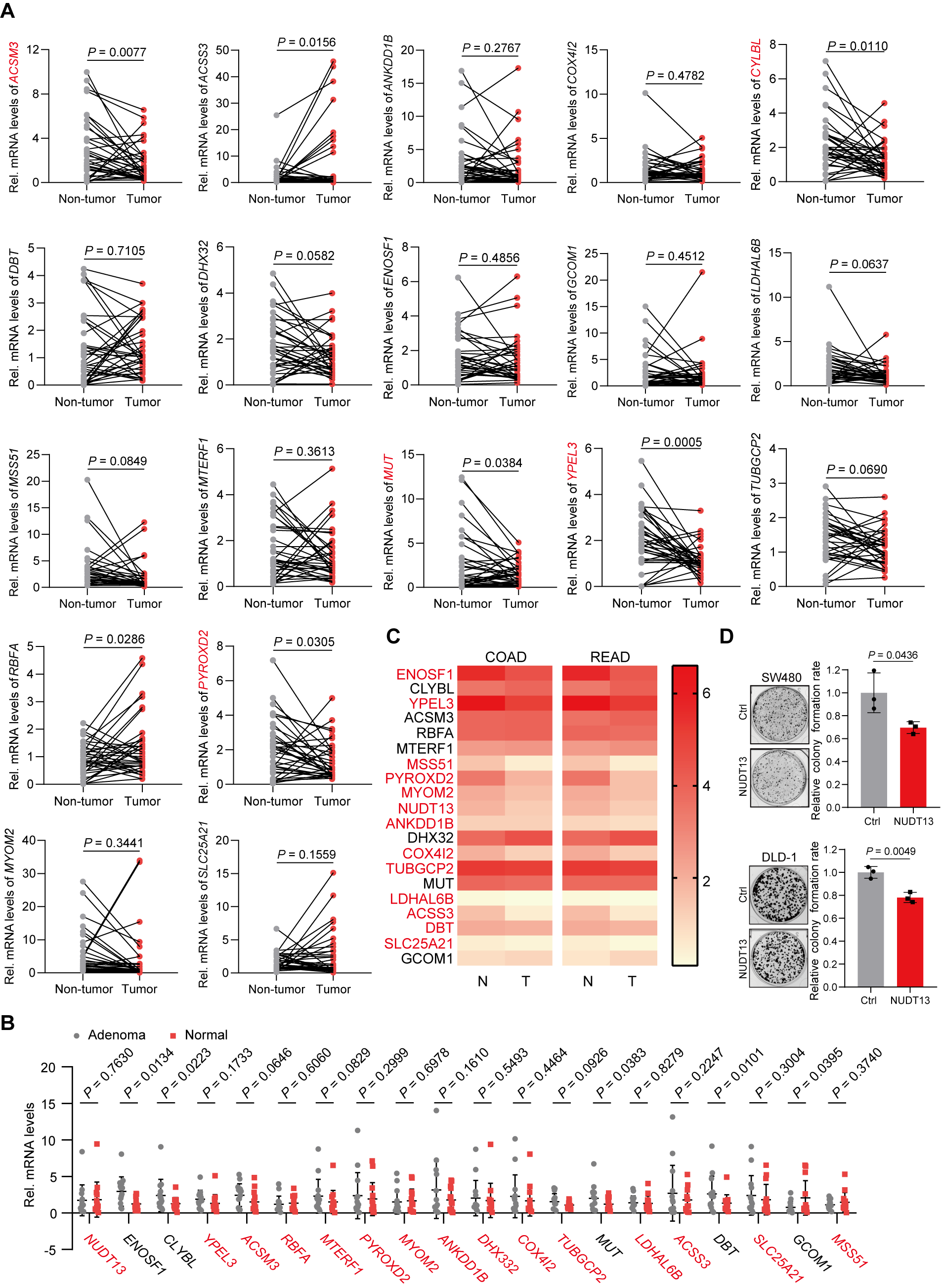


**Figure S2. NUDT13 is a potential tumor suppressor in CRC initiation. (A)** Relative mRNA expression of the other 19 selected genes mentioned in Fig. 1A was measured in early-stage CRC and paired normal tissues (n=36), normalized to ACTB. The genes with consistent expression in comparison to the RNA-seq results were highlighted in red. **(B)** Relative mRNA expression of the 20 selected genes was measured in 14 paired adenomas. The genes with consistent expression in comparison to the RNA-seq results were highlighted in red. **(C)** Heat map analysis of RNA-seq data from UCSC Xena. Differentially expressed genes between normal tissues and tumors were in red font (left), and the density of color in each block represents the median expression value (right). COAD: colon adenocarcinoma (N: 349 cases. T: 275 cases). READ: rectum adenocarcinoma (N: 318 cases. T: 92 cases). N: normal tissues. T: tumors. Data are presented as mean ± SD. **(D)** Colony formation of SW480 and DLD-1 cells after stable transfection of NUDT13 or control vector (left). The number of clones was counted and presented on the right. All results mentioned above were obtained from 3 or more independent experiments. *P* values were calculated by Student’s t test (A, B, and D), and Welch’s t test (C).

**Figure S3**


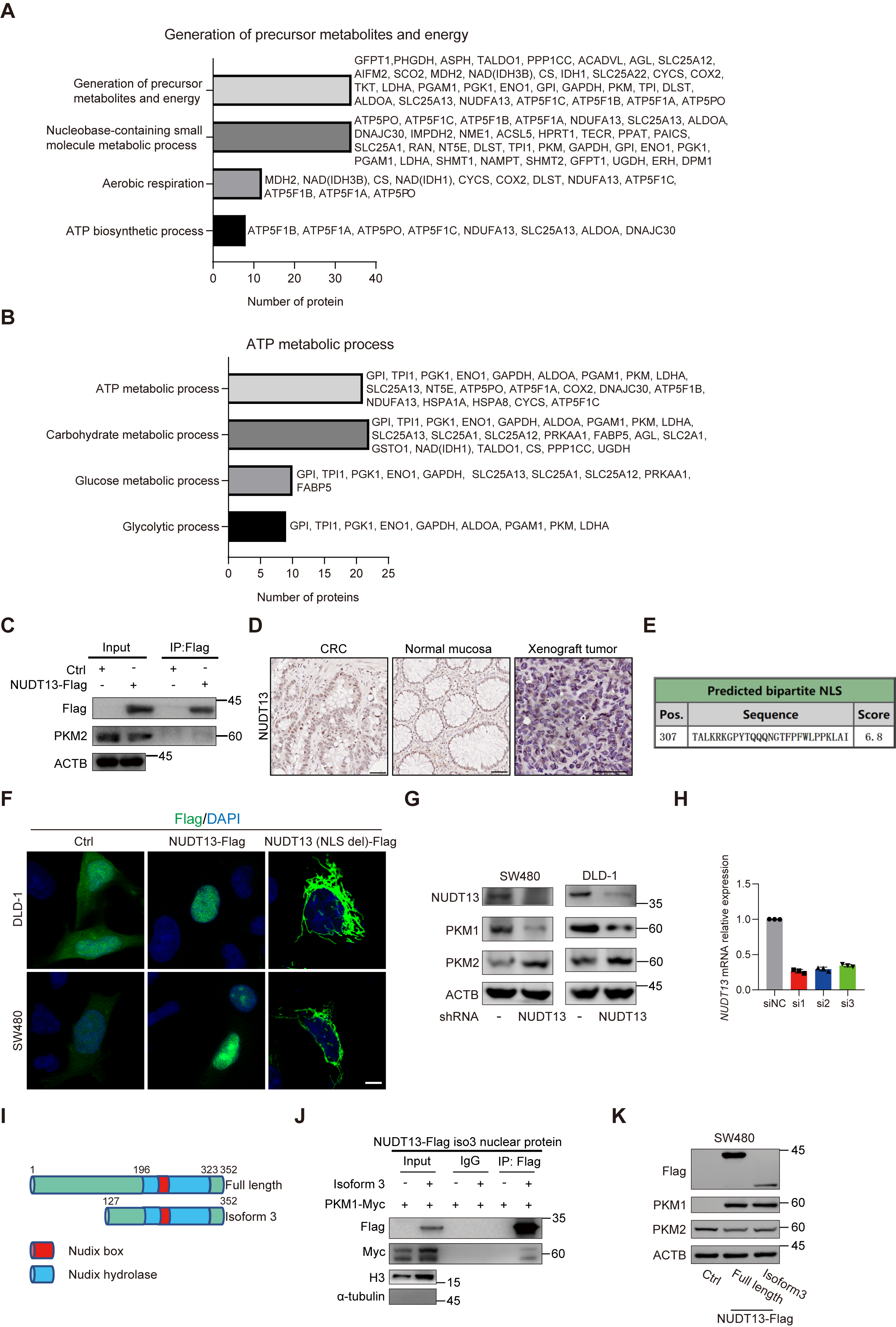


**Figure S3. NUDT13 binds and regulates PKM1. (A and B)** GO biological processes analyses of NUDT13 potential binding partners. **(C)** Co-IP assays showed the unconjugated relation between PKM2 and NUDT13 in SW480 cells. **(D)** IHC results showed the nuclear localization of NUDT13 in CRC, para-tumor tissue, and mouse xenograft tumor. Scale bars, 50 μm. **(E)** The potential NLS sequence within NUDT13, as predicted by NLS Mapper. **(F)** Representative confocal images of DLD-1 and SW480 cells to detect the localization of exogeneous NUDT13-Flag. Scale bars, 20 μm. **(G)** Immunoblot analysis of PKM1 and PKM2 levels after knockdown of NUDT13 in SW480 cells (left) and DLD-1 cells (right). **(H)** Relative *NUDT13* mRNA expression in SW480 after transfection of NUDT13 siRNA or siNC. **(I)** Schematic diagram of NUDT13 isoform 3. **(J)** Co-IP of exogenous NUDT13-Flag isoform 3 and PKM1-myc in nuclear extracts from 293T cells. Histone H3 was used as the nuclear control. **(K)** Immunoblot analysis of PKM1 levels in SW480 transfected with NUDT13-Flag WT or isoform 3 plasmids. All results mentioned above were obtained from 3 independent experiments.

**Figure S4**


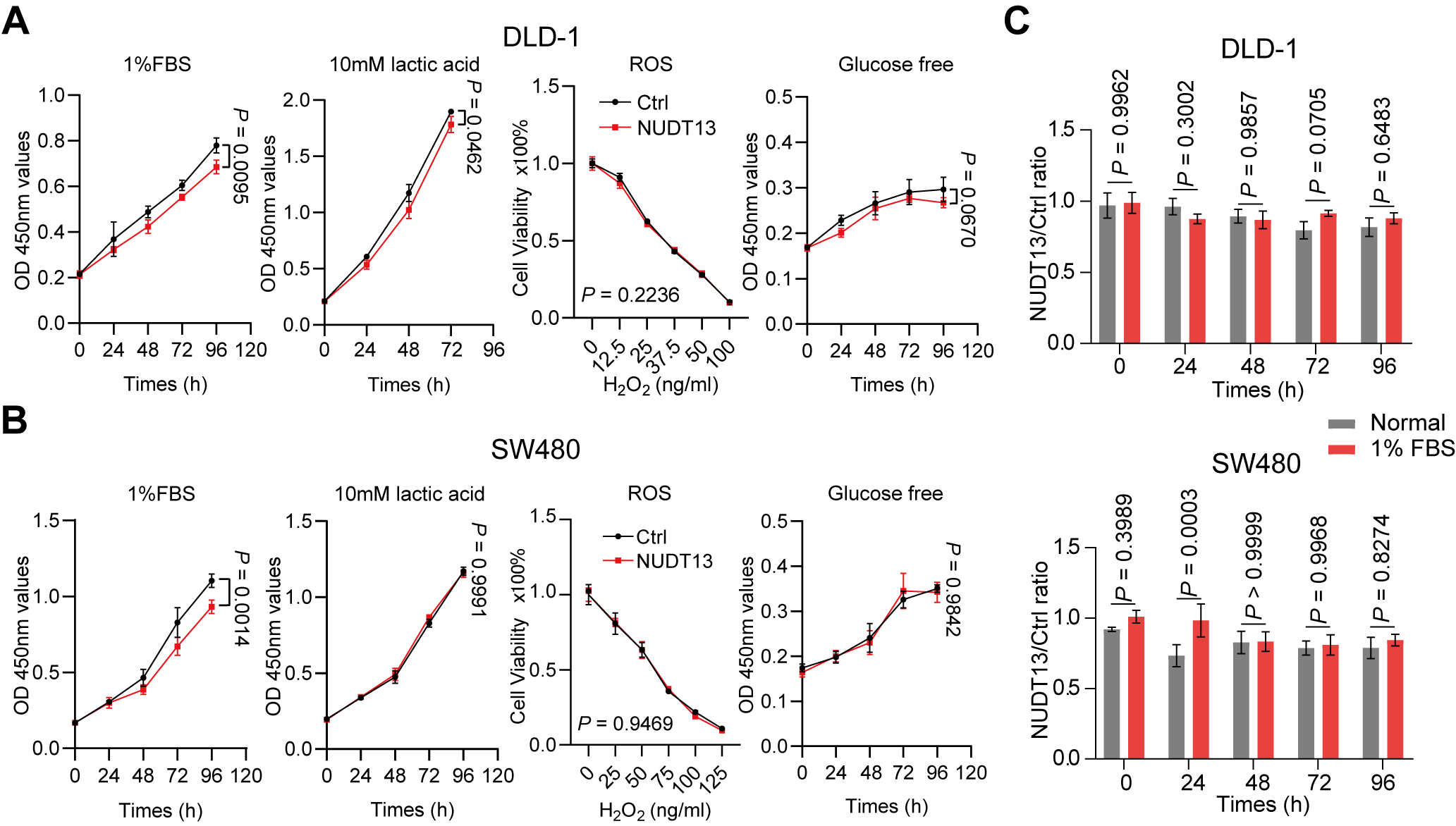


**Figure S4. NUDT13 disturbs the proliferation of tumor cells under hypoxia. (A and B)** The proliferation abilities or cell viability of DLD-1 **(A)** and SW480 cells **(B)** under different stresses, as measured by CCK8. **(C)** The ratio of OD values between NUDT13-overexpressing cells and control cells at the indicated time points under 1% FBS (**A and B**) or normal conditions (**Figure 1F**). All results mentioned above were obtained from 3 or more independent experiments. Data are presented as mean ± SD; *P* values were calculated by two-way ANOVA (A-C).

**Figure S5**


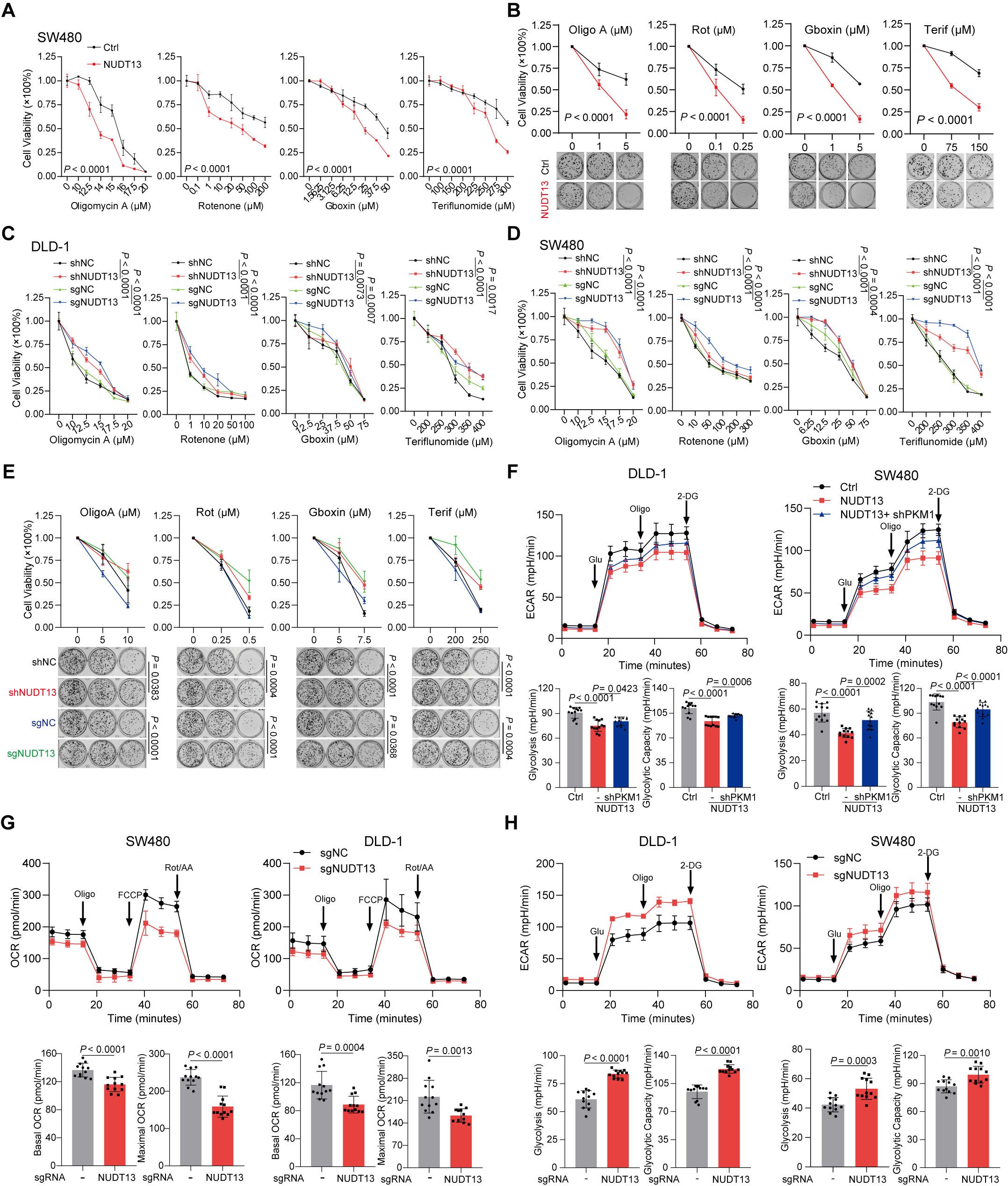


**Figure S5. NUDT13 facilitates OXPHOS phenotype of CRC cells via PKM1.** **(A and B)** The cell viability of NUDT13-overexpressing cells treated with indicated concentrations of Oligomycin A, Rotenone, Gboxin, and Teriflunomide, as measured by CCK8 **(A)** and colony formation assays **(B)**. **(C-E)** The cell viability of NUDT13 knockout or knockdown cells treated with indicated concentrations of Oligomycin A, Rotenone, Gboxin, and Teriflunomide, as measured by as measured by CCK8 **(C and D)** and colony formation assays **(E).** **(F)** Top: the extracellular acidification rates (ECAR) of DLD-1 and SW480 cells transfected with the indicated vectors in response to glucose, oligomycin, and 2-deoxy-D-glucose (2-DG). Bottom: bar graphs depicting the glycolysis (left) and the glycolytic capacity (right) of DLD-1 and SW480 cells. **(G)** Top: the OCR of NUDT13 knockout SW480 and DLD-1 cells in response to oligomycin, FCCP, and rotenone/antimycin A. Bottom: bar graphs depicting the basal OCR (left) and the maximal OCR (right) of SW480 cells and DLD-1 cells. **(H)** The ECAR of NUDT13 knockout DLD-1 and SW480 cells in response to glucose, oligomycin, and 2-DG. Bottom: bar graphs depicting the glycolysis (left) and the glycolytic capacity (right) of DLD-1 and SW480 cells. All results mentioned above were obtained from 3 or more independent experiments. Data are presented as mean ± SD; *P* values were calculated by two-way ANOVA (A-E) and Student’s t test (F-H).

**Figure S6**


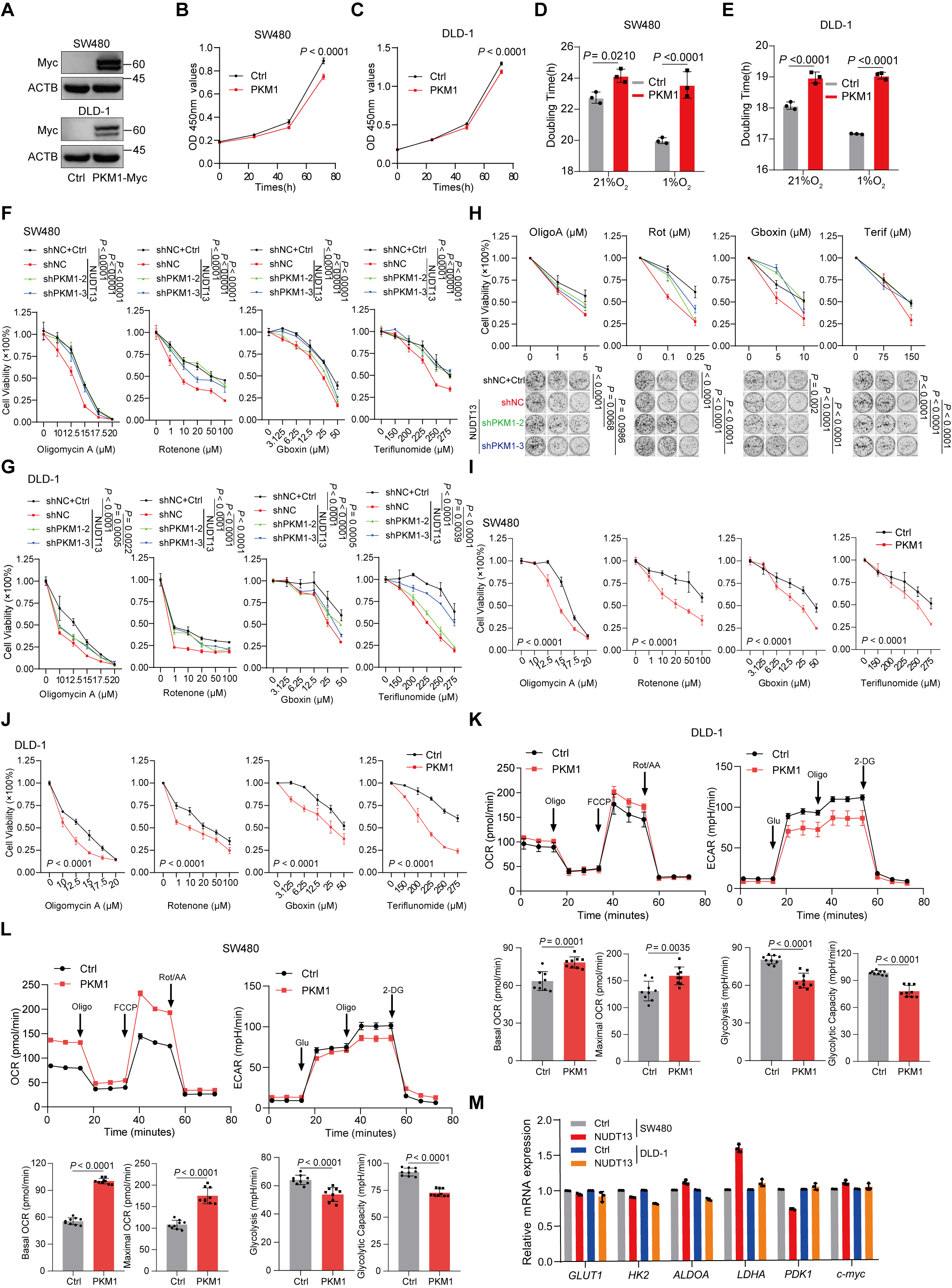


**Figure S6. PKM1 suppresses proliferation ability of CRC cells via enhancing OXPHOS activity. (A-E)** The proliferation abilities of SW480 cells (left) and DLD-1 cells (right) transfected with PKM1, as measured by CCK8 under normoxic or hypoxic conditions. **(F-H)** The cell viability of NUDT13-overexpressing and PKM1 knockdown cells treated with the indicated doses of Oligomycin A, Rotenone, Gboxin, and Teriflunomide, as measured by CCK8 **(F and G)** and colony formation assays **(H)**. **(I and J)** The cell viability of PKM1-overexpressing SW480 **(I)** and DLD-1 cells **(J)** treated with indicated concentrations of Oligomycin A, Rotenone, Gboxin, and Teriflunomide, as measured by CCK8. **(K and L)** The OCR (left) and ECAR (right) of PKM1-overexpressing DLD-1 **(K)** and SW480 cells **(L)**. **(M)** Relative mRNA expressions of several key downstream targets of PKM2, as measured by RT-qPCR. All results mentioned above were obtained from 3 or more independent experiments. Data are presented as mean ± SD; *P* values were calculated by Student’s t test (D, E, K, and L) and two-way ANOVA (B, C, and F-J).

**Figure S7**


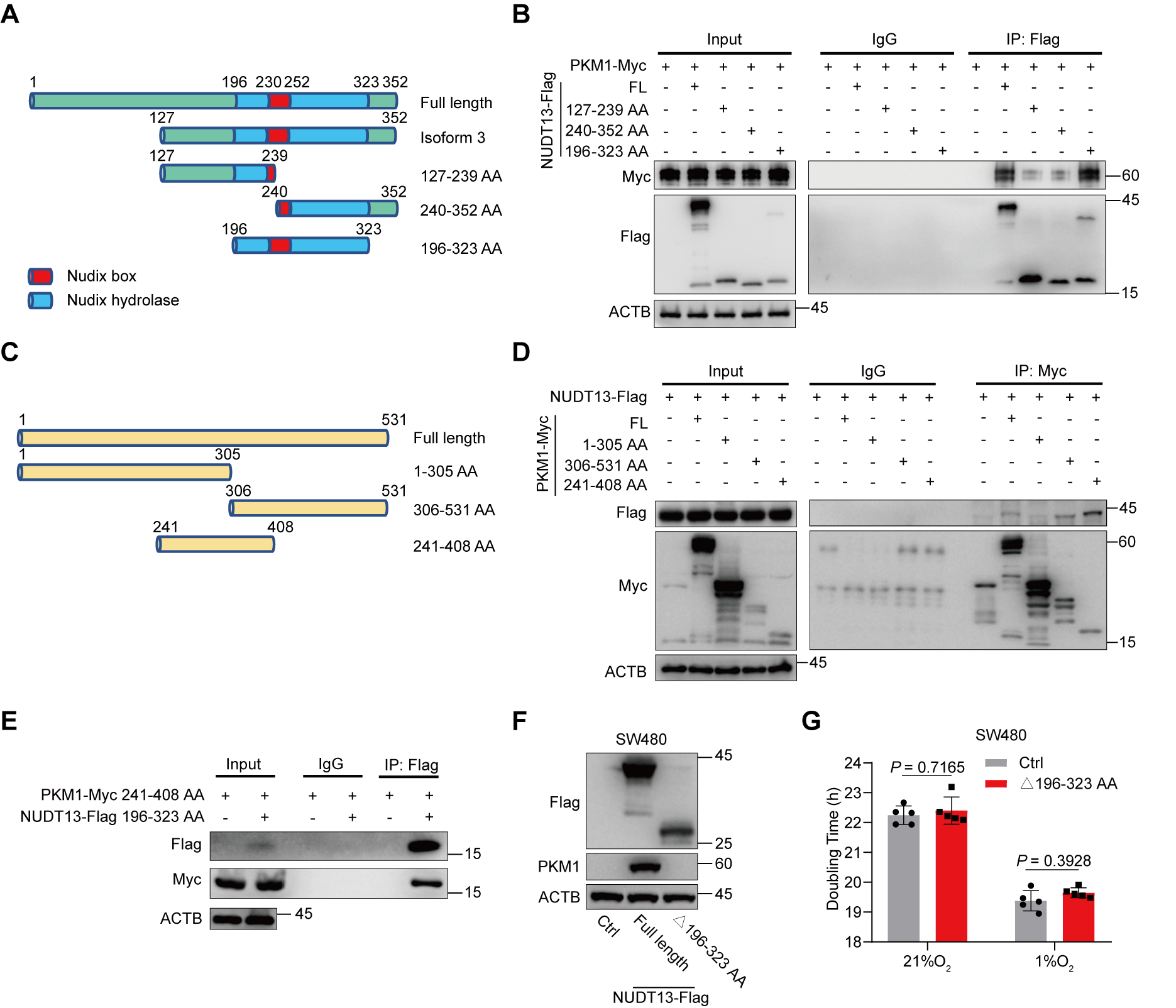


**Figure S7. NUDT13 interacts and regulates PKM1 protein via its NH domain. (A)** Schematic diagram of truncated mutants of NUDT13. **(B)** Co-IP assays were conducted to detect the interaction between PKM1 and NUDT13 using antibody for IgG or flag after co-transfection of plasmids for PKM1-Myc and NUDT13-Flag full-length (FL), residues 127-239, residues 240-352, or residues 196-323 plasmids in 293T cells. **(C)** Schematic diagram of PKM1 truncated mutants. **(D)** Co-IP assay in 293T cells co-transfected with NUDT13-Flag and PKM1-myc FL, residuals 1-305, residuals 306-531, or residuals 241-408 plasmids. **(E)** Co-IP assays conducted in 293T cells co-transfected with plasmids for PKM1-Myc residues 241-408 and NUDT13-Flag residues 196-323. **(F)** Immunoblot analysis of the effect of a NUDT13 truncated mutant on PKM1 protein levels in SW480 cells. **(G)** The doubling time of SW480 cells transfected with a NUDT13 truncated mutant or control vector under normoxic or hypoxic conditions, as measured by CCK8. All results mentioned above were obtained from 3 or more independent experiments. Data are presented as mean ± SD; *P* values were calculated by Student’s t test (G).

**Figure S8**


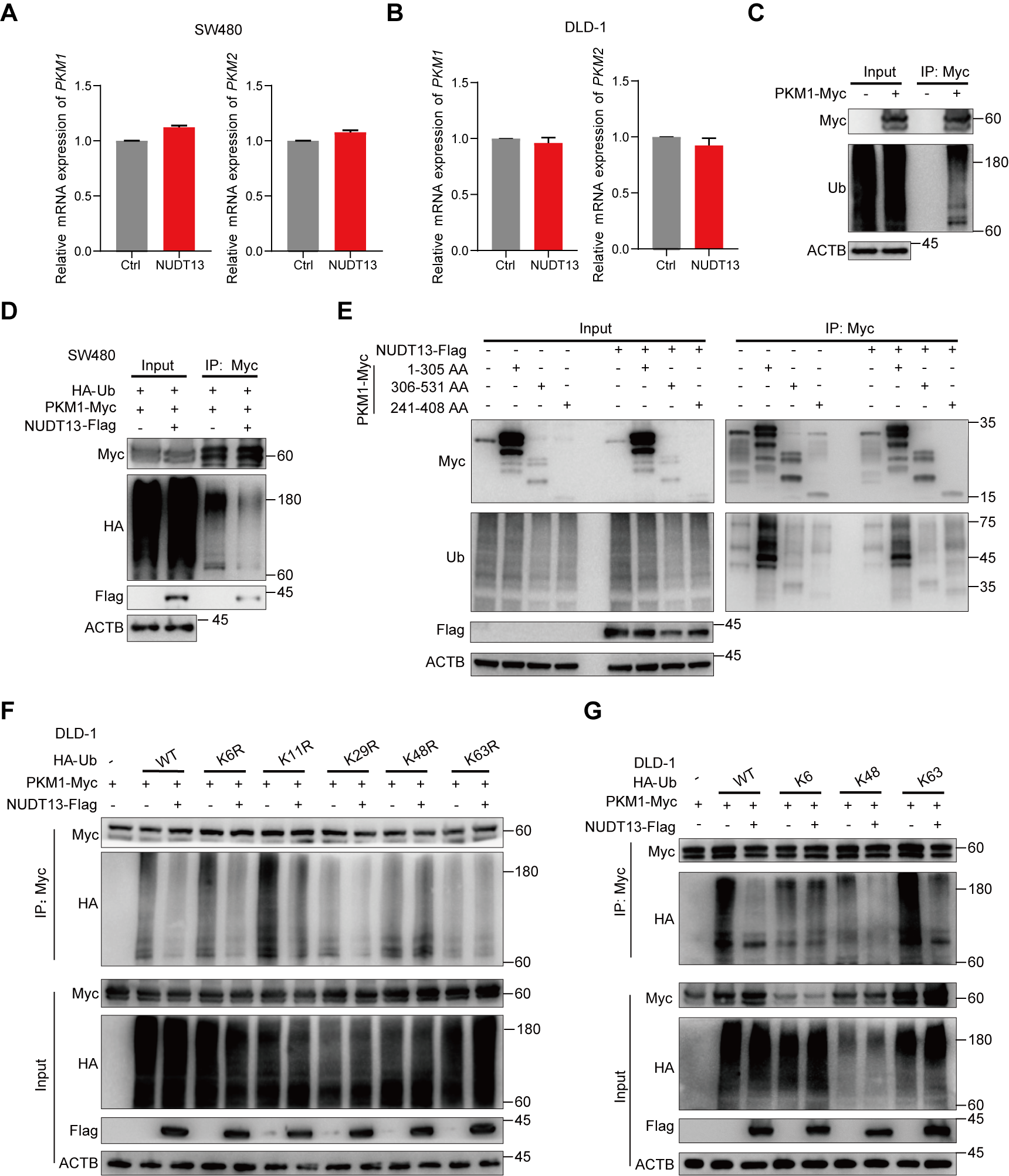


**Figure S8. NUDT13 interacts and stabilizes PKM1 protein by inhibiting its K48- and K63 ubiquitination.** **(A and B)** Relative mRNA expressions of *PKM1* and *PKM2* in SW480 **(A)** and DLD-1 cells **(B)** transfected with NUDT13. **(C)** Immunoblot analysis of exogenous PKM1 ubiquitination level in 293T cells transfected with PKM1-myc. **(D)** Immunoblot analysis of PKM1 ubiquitination levels after upregulation of NUDT13 in SW480 cells transfected with PKM1-myc and HA-Ub. **(E)** Immunoblot analysis of the ubiquitinated part in PKM1 that is affected by NUDT13. **(F and G)** Immunoblot analysis of PKM1 ubiquitin linkage types that are affected by NUDT13 in DLD-1 cells transfected with PKM1-myc, NUDT13-Flag, and HA-Ub WT, or ubiquitin mutants (K6R, K11R, K29R, K48R, K63R, K6, K48 or K63). All results mentioned above were obtained from 3 independent experiments.

**Figure S9**


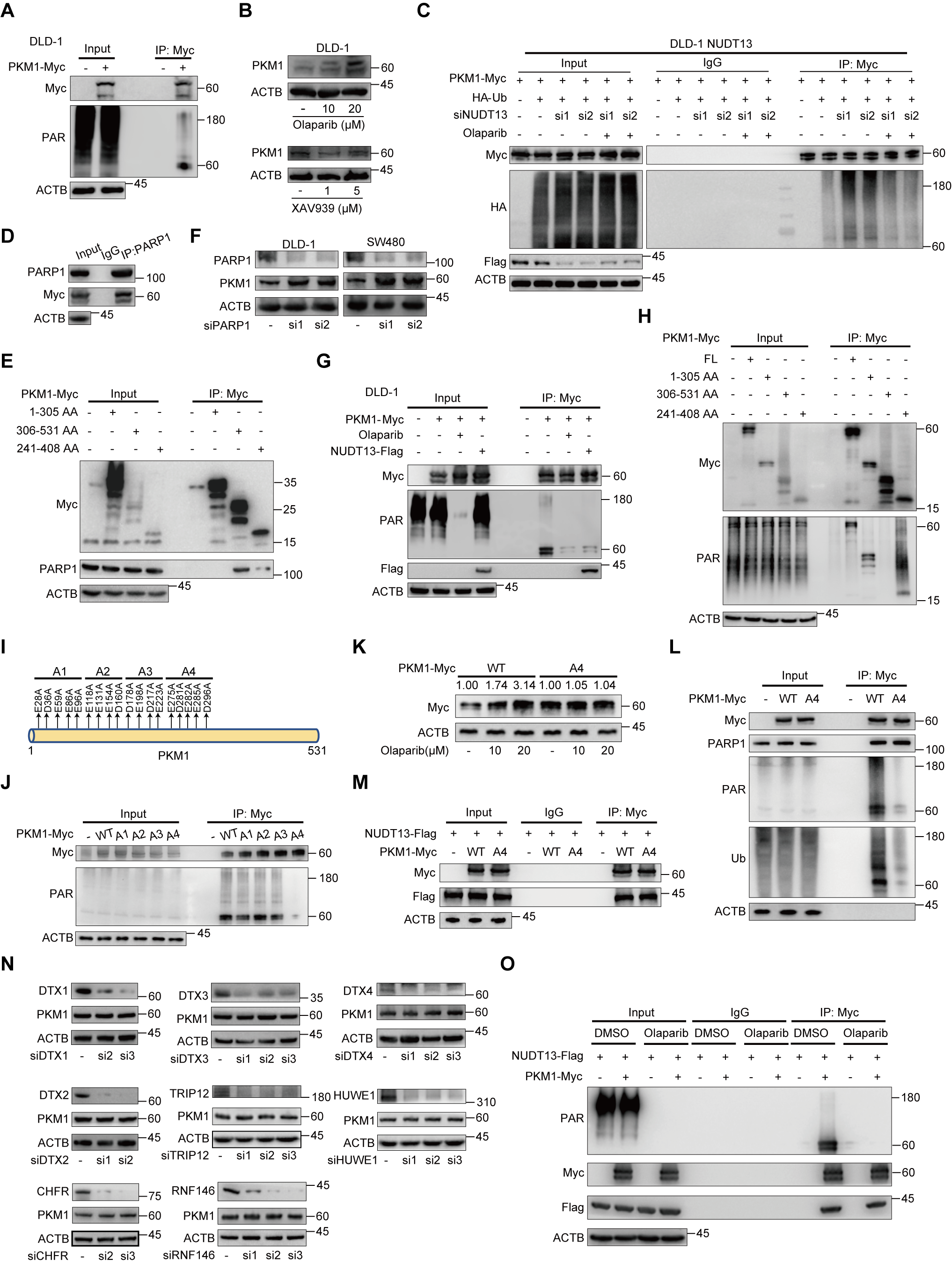


**Figure S9. PARP1 modifies the PARylation of PKM1 protein. (A)** *In vivo* PARylation assay was performed in DLD-1 cells transfected with PKM1-Myc. **(B)** Immunoblot analysis of PKM1 levels in DLD-1 cells treated with indicated doses of Olaparib (48h) or XAV939 (24h). **(C)** Immunoblot analysis of PKM1 ubiquitination levels in NUDT13-overexpressing DLD-1 cells transfected with NUDT13 siRNA and treated with or without Olaparib (10 μM) for 48h. **(D)** Co-IP assay in 293T cells transfected with PKM1-myc. **(E)** Co-IP assay in 293T cells transfected with PKM1-myc residuals 1-305, residuals 306-531, or residuals 241-408 plasmids. **(F)** Immunoblot analysis of PKM1 levels in DLD-1 and SW480 cells transfected with siPARP1. **(G)** Immunoblot analysis of the PKM1 PARylation levels in DLD-1 cells treated with Olaparib (10 μM) for 48h, or transfected with NUDT13-Flag plasmids. **(H)** *In vivo* PARylation assay was performed in 293T cells transfected with PKM1 FL or truncated mutants. **(I)** Schematic diagram of mutation sites on PKM1. **(J)** Immunoblot analysis of PKM1 PARylation levels in 293T cells transfected with WT or mutant PKM1. The amounts of PKM1 levels in different groups were adjusted by MG132 treatment. **(K)** Immunoblot analysis of PKM1 WT or A4 mutant levels in 293T cells treated with the indicated doses of Olaparib for 48h. **(L)** Immunoblot analysis of PKM1 ubiquitination and PARylation levels and the interaction between PKM1 and PARP1 in 293T cells transfected with WT or mutant PKM1. **(M)** Co-IP assays conducted in 293T cells co-transfected with NUDT13-Flag and PKM1-Myc WT or 4A mutant. **(N)** Immunoblot analysis of the PKM1 protein levels in DLD-1 cells after transfection of siRNAs specific for indicated E3 ligases. **(O)** Immunoblot analysis of the interaction between PKM1 and NUDT13 after treatment with Olaparib (10 μM) for 48h. All results mentioned above were obtained from 3 independent experiments.

**Figure S10**


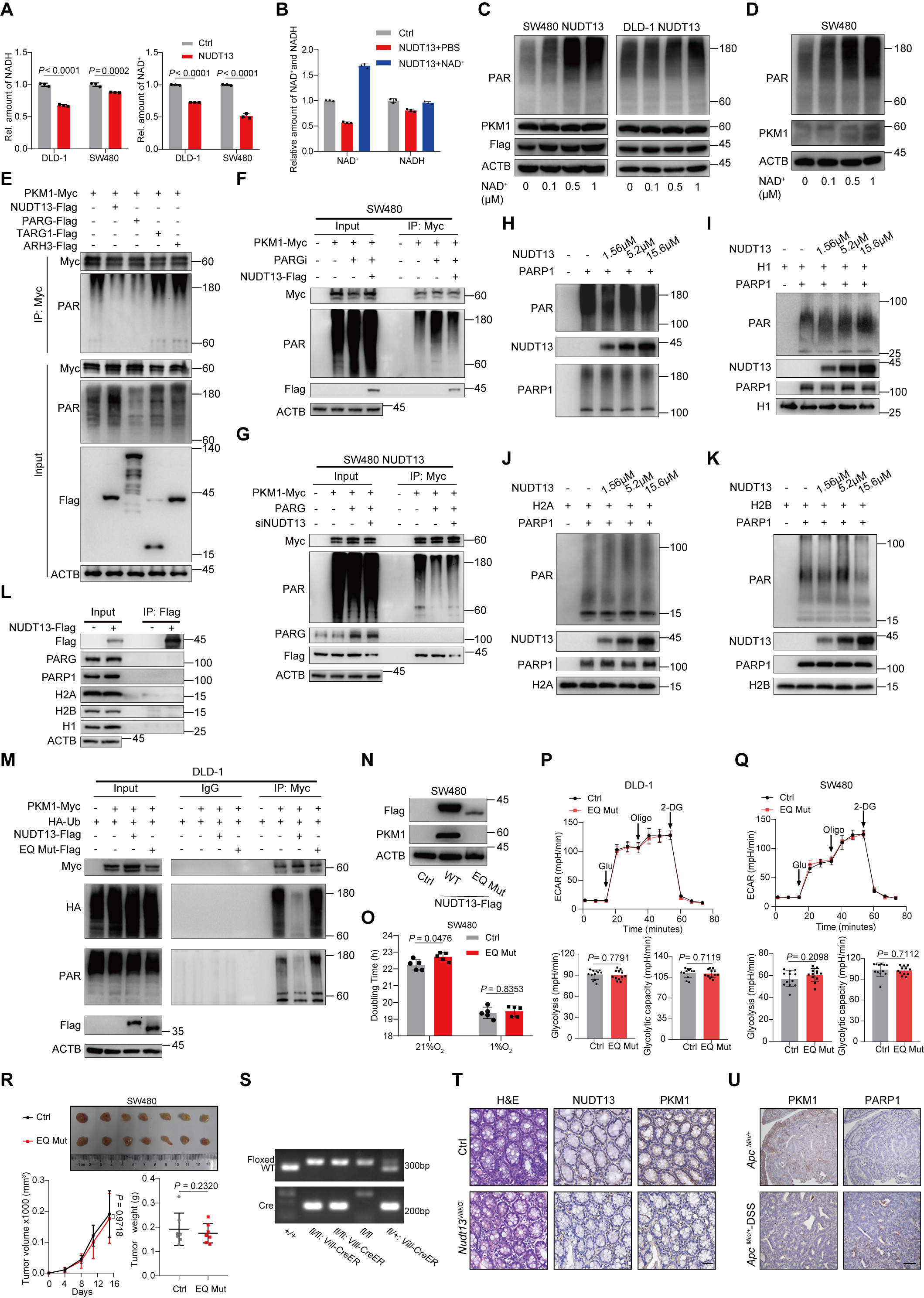


**Figure S10. NUDT13 hydrolase activity is required for PKM1 protein stability. (A)** Cellular NAD^+^ and NADH levels in SW480 and DLD-1 cells after overexpression of NUDT13. **(B-D)** The effect of exogenous indicated doses of NAD^+^ (24h) in cellular NAD^+^/NADH pool **(B)**, PARylation, and PKM1 protein levels **(C and D)**. **(E)** Immunoblot analysis of PKM1 PARylation levels in 293T cells transfected with different ADP-ribosylation erasers. **(F)** Immunoblot analysis of PKM1 PARylation levels in SW480 cells transfected with indicated plasmids, and treated with or without PARGi (10 μM) for 2h before harvest. **(G)** Immunoblot analysis of PKM1 PARylation levels in NUDT13-proficient SW480 cells transfected with indicated plasmids and siRNAs. **(H-K)** Immunoblot analysis of PARylation levels after incubation of PARylated PARP1 **(H)**, histone H1 **(I)**, H2A **(J)**, and H2B **(K)** with control or indicated concentrations of recombinant hNUDT13. **(L)** Co-IP assays showed the unconjugated relations between NUDT13 and the indicated proteins in 293T cells. **(M)** Immunoblot analysis of PKM1 PARylation and ubiquitination levels in DLD-1 cells transfected with NUDT13-Flag or EQ mutant plasmids. **(N)** Immunoblot analysis of the PKM1 level in SW480 cells transfected with NUDT13-Flag WT or EQ mutant plasmids. **(O)** The doubling time of NUDT13 EQ mutant proficient SW480 cells. **(P and Q)** The ECAR of EQ mutant-overexpressing DLD-1 **(P)** and SW480 cells **(Q)** in response to glucose, oligomycin, and 2-DG. Bottom: bar graphs depicting the glycolysis (left) and the glycolytic capacity (right) of DLD-1 **(P)** and SW480 cells **(Q)**. **(R)** Xenograft tumors formed in BALB/c nude mice (n=7). Subcutaneous tumors were measured by volume (left) and weight (right). **(S)** PCR amplification of genomic DNA extracted from transgenic mice using primers against the *Nudt13^flox^* allele and the *Villin-CreERT2* allele as described in Supplementary Table S6. **(T)** H&E, anti-PKM1, and anti-NUDT13 staining of serial colon sections from control (corn oil) and *Nudt13^VillKO^* (tamoxifen) mice. Scale bars, 50 μm. **(U)** IHC detection of PKM1 and PARP1 in mouse CRCs (*Apc^Min/+^*-DSS) and adenomas (*Apc^Min/+^*). Scale bars, 100 μm. All results mentioned above were obtained from 3 or more independent experiments. Data are presented as mean ± SD; *P* values were calculated by Student’s t test (A and O-R) and two-way ANOVA (R).

**Figure S11**


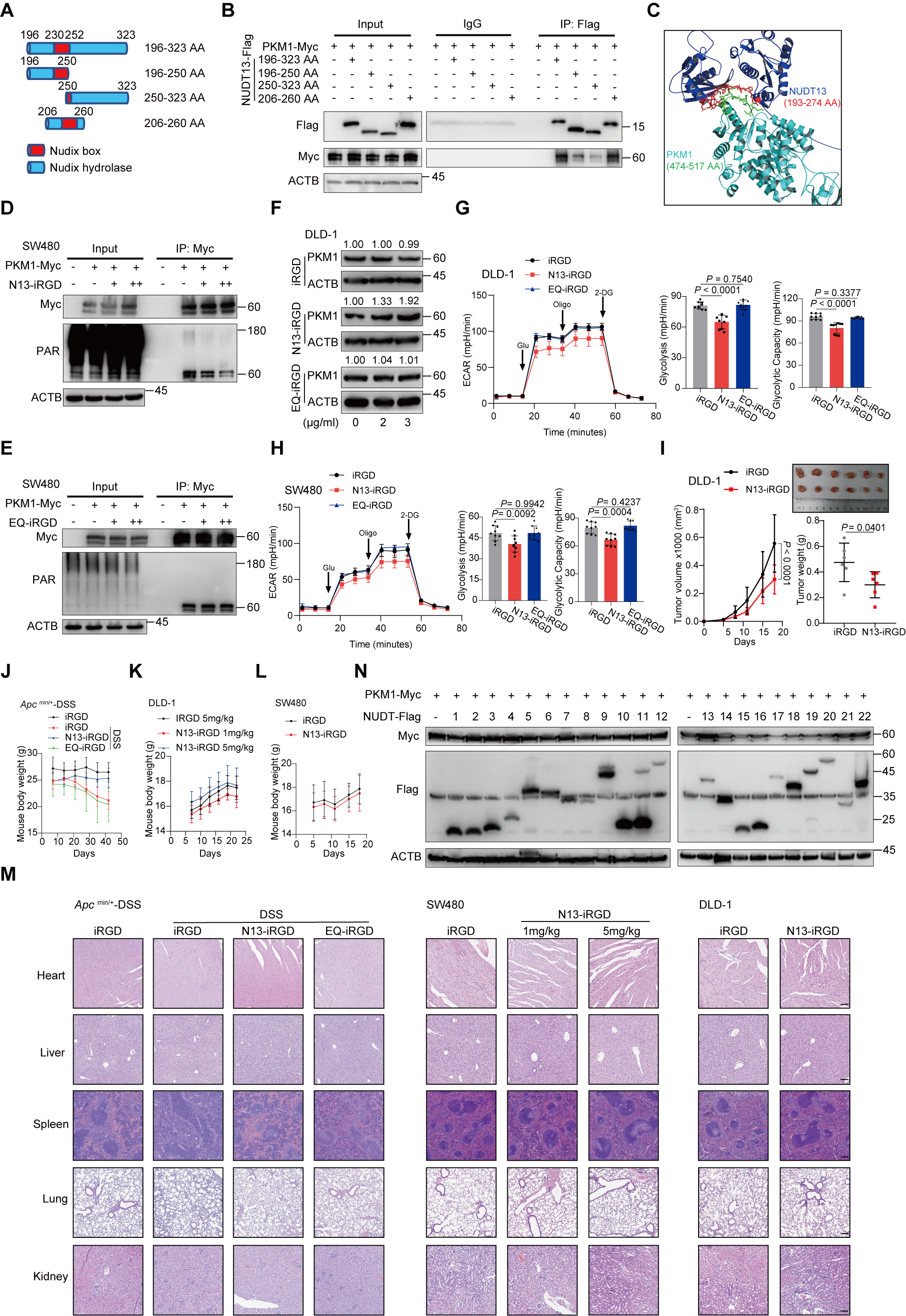


**Figure S11. The NUDT13 residuals 230-252 hold therapeutic prospects. (A)** Schematic diagram of NUDT13 truncated mutants. **(B)** Co-IP assay in 293T cells co-transfected with plasmids for PKM1-myc and NUDT13-Flag residues 196-323, residues 196-250, residues 250-323, or residues 206-260. **(C)** Crystal structures of the predicted docking interfaces between PKM1 (green) and NUDT13 (red). **(D and E)** Immunoblot analysis of PKM1 PARylation level in SW480 cells treated with different doses of N13-iRGD **(D)** or EQ-iRGD **(E)** for 48h. **(F)** Immunoblot analysis of PKM1 protein level in DLD-1 cells treated with indicated doses of fusion peptides for 48h. **(G and H)** The ECAR of DLD-1 cells **(G)** and SW480 cells **(H)** treated with indicated peptides in response to glucose, oligomycin, and 2-DG. Bottom: bar graphs depicting the glycolysis (left) and the glycolytic capacity (right) of DLD-1 cells **(G)** and SW480 cells **(H)**. **(I)** Xenograft tumors formed in BALB/c nude mice (n=6) **(F)**. Subcutaneous tumors were measured by volume (left) and weight (right). **(J-L)** Mouse body weights were measured to assess the side effects of peptide treatment. **(M)** Representative H&E staining of main organs from mice treated with peptide. The first row shows stained sections of normal hearts; myocardial cells are normal in size and structure, and no inflammatory cell infiltration is observed in the myocardial interstitium in all groups. The second row shows stained sections of normal livers; the hepatocytes, portal triads, hepatic plates, and central veins are morphologically normal in all groups. The third row shows stained sections of normal spleens; the size, structure, composition, and ratio of red pulps and white pulps are normal in all treatment groups. The fourth row shows stained sections of normal lungs; the size, structure, and distribution of alveoli, bronchiole terminals, and blood vessels are normal, and pulmonary interstitium is clear and normal. The fifth row shows stained sections of normal kidneys; the glomeruli and tubules are of normal size, structure, and distribution. Scale bars, 100 μm. **(N)** Immunoblot analysis of PKM1 protein level in 293T cells co-transfected with PKM1-myc and NUDT plasmids. All results mentioned above were obtained from 3 or more independent experiments. Data are presented as mean ± SD; *P* values were calculated by Student’s t test (G-I) and two-way ANOVA (I).

**Supporting Tables for**

**Nudix Hydrolase 13 Impairs the Initiation of Colorectal Cancer by Inhibiting PKM1 ADP-Ribosylation**

Jinlong Lin, Yixin Yin, Jinghua Cao, Bingxu Zou, Kai Han, Yufan Chen, Siyu Li, Cijun Huang, Jiewei Chen, Yongrui Lv, Shuidan Xu, Dan Xie, Fengwei Wang

**Table S2. Correlation analysis between NUDT13 protein levels and clinicopathological features of CRC.**

| Variable | Number of cases | NUDT13 level | | *P* Value |
| --- | --- | --- | --- | --- |
|  |  | High  (*N*=87) | Low  (*N*=87) |  |
| **Age, yr** |  |  |  |  |
| ≥60 | 91 | 42 | 49 |  |
| <60 | 83 | 45 | 38 | 0.288 |
| **Gender** |  |  |  |  |
| Female | 69 | 37 | 32 |  |
| Male | 105 | 50 | 55 | 0.439 |
| **Tumor location** |  |  |  |  |
| Colon | 137 | 70 | 67 |  |
| Rectum | 37 | 17 | 20 | 0.578 |
| **pT status** |  |  |  |  |
| T1-T2 | 60 | 40 | 20 |  |
| T3-T4 | 114 | 47 | 67 | **0.001** |
| **pN status** |  |  |  |  |
| N0 | 84 | 40 | 44 |  |
| N1-N2 | 90 | 47 | 43 | 0.544 |
| **pM status** |  |  |  |  |
| M0 | 123 | 62 | 61 |  |
| M1 | 51 | 25 | 26 | 0.867 |
| **Clinical stage** |  |  |  |  |
| I+II | 87 | 53 | 34 |  |
| III+IV | 87 | 34 | 53 | **0.004** |

* χ^2^ text

CRC, colorectal carcinoma;

Statistically significant *P* are in bold (*P*<0.05).

**Table S3. Univariate and multivariate** **Cox regression analysis of different prognostic parameters in CRC patients.**

| Variable | Subset | Hazard ratio for OS (95% CI) | *P Value* |
| --- | --- | --- | --- |
| Univariate analysis (n=174) | | | |
| Age (yr) | ≥60 vs <60 | 0.576 (0.281 - 1.181) | 0.132 |
| Gender | Male vs Femal | 0.568 (0.263 - 1.228) | 0.151 |
| Tomor location | Colon vs Rectum | 0.756 (0.291 - 1.968) | 0.567 |
| pT status | T3+4 vs T1+2 | 1.883 (0.844 – 4.198) | 0.122 |
| pN status | N1+2 vs N0 | 2.191 (1.063 - 4.518) | 0.034 |
| pM status | M1 vs M0 | 7.536 (3.482 - 16.306) | <0.001 |
| Clinical stage | III+IV vs I+II | 5.827 (2.387 - 14.225) | <0.001 |
| NUDT13 level | High vs Low | 0.307 (0.145 - 0.651) | 0.002 |
| PKM1 level | High vs Low | 0.704 (0.348 - 1.425) | 0.330 |
| Multivariate analysis (n=174) | | | |
| pN status | N1+2 vs N0 | 1.403 (0.641 - 3.072) | 0.397 |
| pM status | M1 vs M0 | 6.563 (2.202 - 19.555) | **0.001** |
| Clinical stage | III+IV vs I+II | 1.232 (0.337 - 4.507) | 0.753 |
| NUDT13 level | High vs Low | 0.319 (0.145 - 0.704) | **0.005** |

Statistically significant *P* are in bold (*P*<0.05).

**Table S4. Correlation analysis between PKM1 protein levels and clinicopathological features of CRC.**

| Variable | Number of cases | PKM1 level | | *P** |
| --- | --- | --- | --- | --- |
|  |  | High  (*N*=88) | Low  (*N*=86) |  |
| **Age, yr** |  |  |  |  |
| ≥60 | 91 | 48 | 43 |  |
| <60 | 83 | 40 | 43 | 0.549 |
| **Gender** |  |  |  |  |
| Female | 69 | 37 | 32 |  |
| Male | 105 | 51 | 54 | 0.514 |
| **Tumor location** |  |  |  |  |
| Colon | 137 | 66 | 71 |  |
| Rectum | 37 | 22 | 15 | 0.223 |
| **pT status** |  |  |  |  |
| T1-T2 | 60 | 42 | 18 |  |
| T3-T4 | 114 | 46 | 68 | **<0.001** |
| **pN status** |  |  |  |  |
| N0 | 84 | 46 | 38 |  |
| N1-N2 | 90 | 42 | 48 | 0.286 |
| **pM status** |  |  |  |  |
| M0 | 123 | 67 | 56 |  |
| M1 | 51 | 21 | 30 | 0.110 |
| **Clinical stage** |  |  |  |  |
| I+II | 87 | 52 | 35 |  |
| III+IV | 87 | 36 | 51 | **0.015** |

* χ^2^ text

CRC, colorectal carcinoma;

Statistically significant *P* are in bold (*P*<0.05).

**Table S5. The antibodies used in this study**

| **Name** | **Manufacturer** | **Lot.No.** | **Source** | **Application** | **RRID** |
| --- | --- | --- | --- | --- | --- |
| NUDT13 | Invitrogen | PA569554 | Rabbit | WB/IHC/IF | AB_2689237 |
| PKM1 | Proteintech | 15821-1-AP | Rabbit | WB/IP/IHC | AB_2163820 |
| PKM2 | Proteintech | 15822-1-AP | Rabbit | WB | AB_1851537 |
| PAR/pADPr | R&D systems | 4335-MC-100 | Mouse | WB | AB_2572318 |
| PARP1 | CST | 9532S | Rabbit | WB/IP | AB_659884 |
| PARP2 | Proteintech | 55149-1-AP | Rabbit | WB | AB_10858796 |
| Ub | CST | 3936S | Mouse | WB | AB_331292 |
| Flag | sigma | F1804 | Mouse | WB/IP/IF | AB_262044 |
| Flag | CST | 14793S | Rabbit | WB | AB_2572291 |
| myc | Proteintech | 16286-1-AP | Rabbit | WB/IP | AB_11182162 |
| myc | CST | 2276S | Mouse | WB | AB_331783 |
| HA | CST | 3724S | Rabbit | WB/IP | AB_1549585 |
| ACTB | CST | 3700S | Mouse | WB | AB_2242334 |
| Histone H3 | Proteintech | 17168-1-AP | Rabbit | WB | AB_2716755 |
| α-tubulin | Proteintech | 66031-1-lg | Mouse | WB | AB_11042766 |
| RNF146 | Abcam | Ab201212 | Rabbit | WB |  |
| HUWE1 | Proteintech | 19430-1-AP | Rabbit | WB | AB_2878579 |
| CHFR | Proteintech | 12169-1-AP | Rabbit | WB | AB_2080821 |
| TRIP12 | Proteintech | 25303-1-AP | Rabbit | WB | AB_2880020 |
| DTX1 | Proteintech | 18350-1-AP | Rabbit | WB | AB_2878536 |
| DTX2 | Proteintech | 18565-1-AP | Rabbit | WB | AB_10643380 |
| DTX3 | Proteintech | 25304-1-AP | Rabbit | WB | AB_2880021 |
| DTX4 | Proteintech | 25222-1-AP | Rabbit | WB | AB_2879970 |
| PARG | Proteintech | 27808-1-AP | Rabbit | WB | AB_2880979 |
| Histone H1 | Proteintech | 18201-1-AP | Rabbit | WB | AB_10859820 |
| Histone H2A | Immunoway | YT5500 | Rabbit | WB |  |
| Histone H2B | Proteintech | 15857-1-AP | Rabbit | WB | AB_10664929 |

**Table S6. The primer sequences used for RT-PCR**

| **Gene** | **Forward primer 5’-3’** | **Reverse primer 5’-3’** |
| --- | --- | --- |
| NUDT13 | ATCACGCTGGTGTCAGATGG | AGTACTGCAGGCTTTCCACC |
| PKM1 | CAGCCAAAGGGGACTATCCT | GAGGCTCGCACAAGTTCTTC |
| PKM2 | CTATCCTCTGGAGGCTGTGC | GTGGGGTCGCTGGTAATG |
| c-myc | GGCTCCTGGCAAAAGGTCA | CTGCGTAGTTGTGCTGATGT |
| GLUT1 | TGGCATCAACGCTGTCTTCT | AACAGCGACACGACAGTGAA |
| HK2 | GAGCCACCACTCACCCTACT | ACCCAAAGCACACGGAAGTT |
| ALDOA | CGAGAACACCGAGGAGAACC | ACACCGCCCTTGGATTTGAT |
| LDHA | ATGGCAACTCTAAAGGATCAGC | CCAACCCCAACAACTGTAATCT |
| PDK1 | CTGTGATACGGATCAGAAACCG | TCCACCAAACAATAAAGAGTGCT |
| ACTB | CCAACCGCGAGAAGATGA | TCCATCACGATGCCAGTG |
| ENOSF1 | AAGCCTGTCTGGAAGTTACTTGT | GCAGGGTATCCTTGTGCCA |
| CLYBL | TCCCCAGACTTGGATATAGTTCC | TGCACAATCTACATTCAGGGATG |
| YPEL3 | GTGCGGATTTCAAAGCCCAAG | CCCACGTTCACCACTGAGTT |
| ACSM3 | AGGAAGATGCTACGTCATGCC | ATCCCCAGTTTGAAGTCCTGT |
| RBFA | TTGGGTTCTCACTCGACTTACA | GACTCACTTCAGGGGTACACA |
| MTERF1 | TTGGATGACTCGATTTTCAGCA | GCTGTCGTTTCCTTGCCAT |
| MSS51 | GGCTTTGGATTTCGATGTCCT | CTAGGGAGTGCTCTACAGTGAG |
| PYROXD2 | CATCGCTTGGCATTAGCCATT | GAGGACCTCATAATATCGGGGA |
| MYOM2 | GAGAGACACACATTTGAAGAGCG | TCCTGTACTTTCCCGGTTCAG |
| ANKDD1B | CACATTCGCACGCTTCTCTG | CCTGCGTGTACCAGCTCTTC |
| DHX32 | GTTCAAGCCACTCGATGAAACA | TGACTGAGTTGCTCCAGATCAA |
| COX4I2 | ACTACCCCATGCCAGAAGAG | TCATTGGAGCGACGGTTCATC |
| TUBGCP2 | CTTCCTTCGAGTACGGGCAG | GGCTGGCTGGATGTAGAACC |
| MUT | AGAAGACCTAATATGGCACACCC | ATGGTCCAGGGCCTAAAGGTA |
| LDHAL6B | AAGCGGCTGTAATCTGGATACT | ACCAGCTATGTTCACTCCACT |
| DBT | CTCCGGTATTCACAGGCAAAG | AAAATGAGGTATCTTCAGGGCTG |
| SLC25A21 | CCAAGCCTGAAGTCAGCTTAG | TGCACATCTCTGAATCTGAAACC |
| GCOM1 | AAGTCAAGCAGATGGTCGAGG | CAATGGCAGCTTTCAGTTTGGC |
| ACSS3 | TGGACCAAAACGCTGGAGAAC | ACGATCAACGGCATTGTAACA |
| *Nudt13^flox^* | GGCTATTCTTATTGAGACCTCCACT | TCGCTTATATGGAACAAGGCAGTA |
| *Villin-CreERT2* | GAACGCACTGATTTCGACCA | GCTAACCAGCGTTTTCGTTC |

**Table S7. The oligonucleotide sequence of siRNAs, shRNAs, and sgRNA.**

| **genes** | **sequence** |
| --- | --- |
| siNUDT13-1 | GGCAGGTTTTTGTGATATA |
| siNUDT13-2 | CCATGCAACTGTGAAACCA |
| siNUDT13-3 | GCAGGAAGCATGGTTTGCT |
| shNUDT13 | CCGGGCAGGAAGCATGGTTTGCTCTCGAGAGCAAACCATGCTTCCTGCTTTTTT |
| sgNUDT13 | CACCGTGTGCTGAAGTCTGAAGCAG |
| siPARP1-1 | CCATTGAGCACTTCATGAA |
| siPARP1-2 | GATTTCATCTGGTGTGAAT |
| shPKM1-2 | CCGGGCAAGCTGTTTGAAGAACTCTCGAGAGTTCTTCAAACAGCTTGCTTTTTT |
| shPKM1-3 | CCGGAGCCTCAAGTCACTCCACACTCGAGTGTGGAGTGACTTGAGGCTTTTTTT |
| siDTX1-2 | CGAGGATGTGGTTCGAAGA |
| siDTX1-3 | CCATCCGCATCGTCTATGA |
| siDTX2-1 | GGACCATCCTCATAGTTTA |
| siDTX2-2 | AGGGAAAGATGGAGGTATT |
| siDTX3-1 | GAGCGAAGGGTATCACAGA |
| siDTX3-2 | GGATGCTGGTCTCTAAGGA |
| siDTX3-3 | GTGGGACTTCCTGAGCAAA |
| siDTX4-1 | GCTACGTAATTGACTTCAA |
| siDTX4-2 | CAGACACCGTCATCTGGAA |
| siDTX4-3 | GGATCGACCTCACTTCCAT |
| siRNF146-1 | GATGGACAGTGCACAGTAA |
| siRNF146-2 | GTATGTCGCTGATCTTGAA |
| siRNF146-3 | CCGTAAACCTAGCAAGAGA |
| siCHFR-2 | GAAGCAGACATGCCCTTTA |
| siCHFR-3 | CGTGGAGCGGATCTGTAAA |
| siHUWE-1 | GCTCAATACTAGCCGTCTA |
| siHUWE-2 | GAGGCTAAATGTCTAGAGA |
| siHUWE-3 | CAGTGTTGCTCCTGATTGA |
| siTRIP12-1 | GGGCCATGTTAGAAATCCA |
| siTRIP12-2 | GAAGGCGTTTCTAGGCAAT |
| siTRIP12-3 | GCACCTAGATTGGATAGAA |
